# Bioactive recombinant human oncostatin M for NMR-based screening in drug discovery

**DOI:** 10.1038/s41598-021-95424-6

**Published:** 2021-08-10

**Authors:** Olga A. Mass, Joseph Tuccinardi, Luke Woodbury, Cody L. Wolf, Bri Grantham, Kelsey Holdaway, Xinzhu Pu, Matthew D. King, Don L. Warner, Cheryl L. Jorcyk, Lisa R. Warner

**Affiliations:** 1 Biomoleculer Research Center, Boise State University, Boise, ID 83725 USA; 2 Department of Chemistry and Biochemistry, Boise State University, 1910 University Dr., Boise, ID 83725 USA; 3 Biomolecular Sciences Graduate Program, Boise State University, Boise, ID 83725 USA; 4 Department of Biological Sciences, Boise State University, Boise, ID 83725 USA

**Keywords:** Drug screening, Solution-state NMR, Cytokines, Oncostatin M

## Abstract

Oncostatin M (OSM) is a pleiotropic, interleukin-6 family inflammatory cytokine that plays an important role in inflammatory diseases, including inflammatory bowel disease, rheumatoid arthritis, and cancer progression and metastasis. Recently, elevated OSM levels have been found in the serum of COVID-19 patients in intensive care units. Multiple anti-OSM therapeutics have been investigated, but to date no OSM small molecule inhibitors are clinically available. To pursue a high-throughput screening and structure-based drug discovery strategy to design a small molecule inhibitor of OSM, milligram quantities of highly pure, bioactive OSM are required. Here, we developed a reliable protocol to produce highly pure unlabeled and isotope enriched OSM from *E. coli* for biochemical and NMR studies. High yields (ca. 10 mg/L culture) were obtained in rich and minimal defined media cultures. Purified OSM was characterized by mass spectrometry and circular dichroism. The bioactivity was confirmed by induction of OSM/OSM receptor signaling through STAT3 phosphorylation in human breast cancer cells. Optimized buffer conditions yielded ^1^H, ^15^N HSQC NMR spectra with intense, well-dispersed peaks. Titration of ^15^N OSM with a small molecule inhibitor showed chemical shift perturbations for several key residues with a binding affinity of 12.2 ± 3.9 μM. These results demonstrate the value of bioactive recombinant human OSM for NMR-based small molecule screening.

## Introduction

Oncostatin M (OSM) is a pleiotropic inflammatory cytokine and a member of the interleukin-6 (IL-6) family^1^. OSM plays physiological roles in wound healing^2^, viral immune response^3^, hematopoiesis and liver homeostasis^4^, lipid metabolism^5^, and bone development^6^. It also is important in chronic inflammatory diseases such as inflammatory bowel disease^7^ and rheumatoid arthritis^8^. Most recently, OSM was identified as one of the proteins that are found at high levels in the serum of COVID-19 intensive care unit (ICU) patients that correlated with disease severity, potentially pointing to a role for OSM in cytokine storm reactions during SARS-CoV-2 infections^9^.

OSM also plays an important role in cancer invasion and metastasis, specifically in the context of breast cancer^10^, hepatocellular cancer^11^, and prostate carcinomas^12^, to name a few. Our work in defining the role of OSM in breast cancer metastasis has shown that OSM signaling increases tumor cell detachment in a COX2-dependent fashion^13^, epithelial-mesenchymal transition^14^, and the invasive potential of human and mouse mammary carcinoma cells^13–15^. We have further shown that OSM promotes the expression of proteases that can contribute to metastasis^14^ as well as other metastasis-related proangiogenic proteins such as vascular endothelial growth factor (VEGF)^16^ and hypoxia-inducible factor-1α (HIF1α)^16,17^. Finally, we have demonstrated that OSM induces osteolytic bone metastases *in vivo*^18^, as well as lung and other metastases in two different mouse models of breast cancer^19^. Moreover, increased expression of both OSM and the OSM receptor (OSMRβ) have been correlated with increased recurrence of tumors and decreased survival in breast cancer patients^18,20^.

The ability of OSM to stimulate multiple signaling pathways is responsible for the observed pleiotropic effects of OSM in various diseases as well as biological processes. Extracellular OSM stimulates signal transduction through interactions with two unique receptors: the leukemia inhibitory factor receptor (LIFR) and the OSM receptor (OSMR). OSM binds to the heterodimeric OSMR, which is comprised of OSMRβ and gp130, with higher affinity than it binds the heterodimeric LIFR, which consists of **LIFRβ and the common gp130^21^. For example, ERK1/2, PI3K, and STAT1/3 can be activated by OSM binding the LIFR or OSMR, but only signaling through the OSMR can activate STAT5, p38, JNK, and possibly PKC-δ and STAT6^22^.

OSM has been identified as a potential therapeutic target for treatment of inflammatory diseases. Multiple approaches to inhibit OSM signaling have been investigated, including RNA aptamers^23^ and antibody therapies^24^. Specific inhibition of OSM-OSMRβ interactions by neutralizing antibodies targeting the OSM receptor subunit OSMRβ has led to reduced lethality in a mouse model of inflammatory cardiomyopathy^25^ and reduced invasion, angiogenesis, and metastasis in squamous cell carcinoma^26^.

Several challenges exist with the current antibody therapy approach. The slow clearance of antibody therapies from circulation exacerbates this potential issue, especially in cases where a patient sustains an injury that requires an OSM-induced inflammatory response, requires surgical intervention, or contracts an infection. Specifically, GSK2330811 was found to have a plasma elimination half-life of 19–25 days^24^, while small molecule drugs generally have half-lives on the order of hours, which can be of significant clinical benefit in the case of drugs that suppress the inflammatory response^27^. Beyond offering cost advantages for development, there are multiple logistical advantages to orally bioavailable small molecule therapies. These include longer shelf-life and greater stability that precludes the need for refrigeration, which is especially important in rural and other areas that may also have limited experience or infrastructure for parenteral drug administration^27^. A small molecule inhibitor of the OSM-OSMRβ interaction would provide specific disruption of the OSM-specific cell signaling pathway, and a structure-based rational design of small molecule inhibitors uniquely targeting the OSM-OSMRβ interaction could provide a potential treatment for a wide variety of OSM-mediated diseases. Given the extensive role of OSM in inflammatory diseases, cancer, and the potential role of OSM in COVID-19, small molecule inhibitors of OSM could provide a much-needed tool for clinicians.

**Figure 1:**
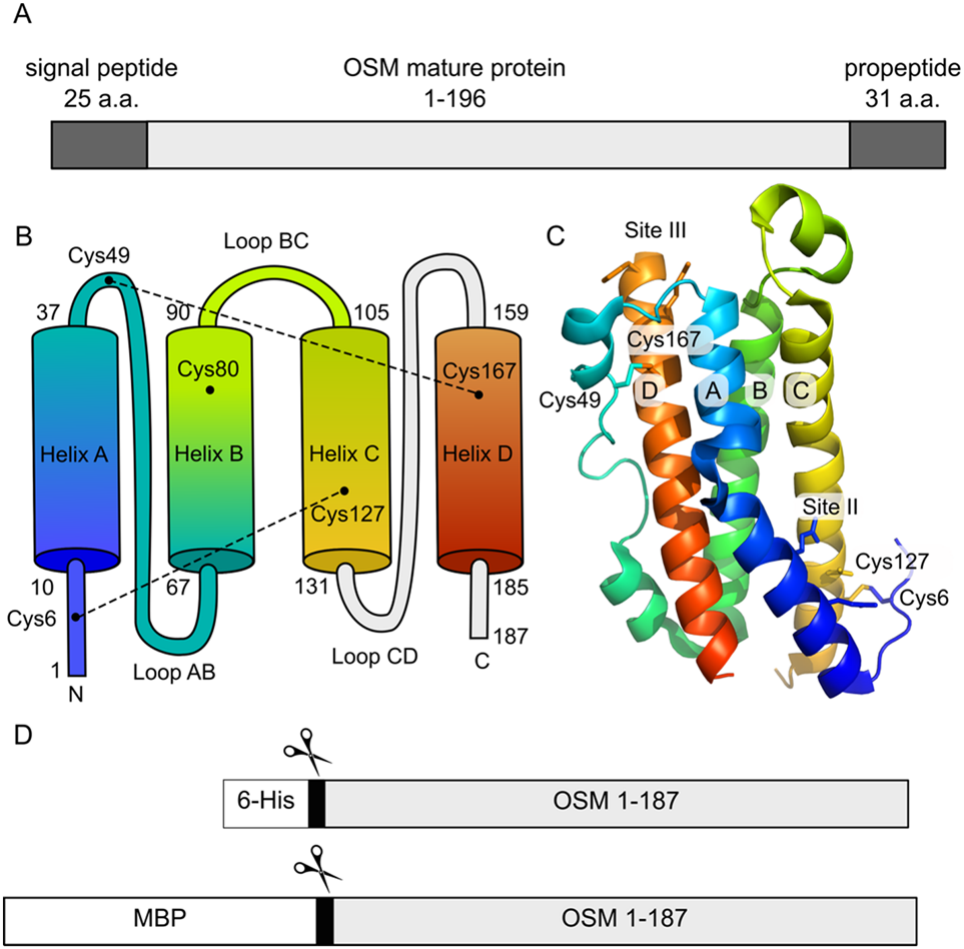
OSM structure overview. (**A**) Schematic representation of the OSM gene product. Signal peptide and propeptide are removed post-translationally. (**B**) Domain architecture of OSM showing the four helices A, B, C and D and disulfide bonds. The Loop CD and the C-terminus were not observed in the crystal structure^28^ and are colored gray. (**C**) Cartoon representation of OSM from PDBID 1EVS, Site II, Site III, and disulfides are shown as sticks, colored according to domain architecture. (**D**) Schematic representation of the expression constructs used in this study. A Tobacco Etch Virus protease site (scissors) was engineered into pD441-NH (6-His) and pD441-MBP (maltose binding protein) fusion constructs that included OSM 1–187.

Although the structure of the OSM-OSMRβ complex remains unsolved, a crystal structure of OSM and alanine scanning assays have provided key insights into how OSM binds to the OSMRβ^28^. The human OSM gene is translated as a 252 amino acid polypeptide, where the first 25 residues are an extracellular secretory signal peptide^29^, and the C-terminal 31 residues are proteolytically cleaved to yield an active 196 residue mature protein^30^ (Fig. 1A). OSM is a four-helix bundle comprised of helices A, B, C and D that are arranged in an up-up-down-down configuration, joined by loops AB, BC and CD and anchored by two intramolecular disulfide bonds (Figs. 1B and 1C). Two receptor binding sites (II and III) are noted in OSM (Fig. 1C). Site II residues Gln20, Gln16, Gly120, and Asn124 are important for OSM interaction with the gp130 subunit of the OSMR^28^. The N-terminal region of helix D contains protruding Phe160 and Lys163 residues that make up an FXXK motif in binding site III that has been shown to be essential for OSM interaction with LIFR, LIFR/gp130, and OSMR/gp130 as measured by receptor binding and cell survival assays^28^. The FXXK motif is thought to be especially important for direct interaction of OSM with the OSMRβ subunit of the OSMR^31^.

A structure-guided approach to designing chemical inhibitors for OSM is needed to specifically inhibit the OSM signaling pathway. Solution nuclear magnetic resonance (NMR) spectroscopy is a particularly useful method for structure-based drug discovery^32,33^. However, NMR is an inherently insensitive technique that requires milligram quantities of isotopically labeled proteins. Thus, a reliable protocol to produce large quantities of highly pure, bioactive recombinant human OSM (rhOSM) is required to fully employ an NMR based structure-based drug design approach and to screen for small molecule ligands in the pursuit of small molecule inhibitors.

Here, we report an efficient protocol for the expression of recombinant, soluble, isotopically labeled rhOSM in high yield and purity in a bacterial expression platform. Prior work by Nguyen et al., described the production of recombinant human OSM using MBP-tagged OSM in SHuffle *E. coli* cells. We detail here our independent findings, including additional examination of the effects of growth medium, cell strains, temperature, construct design, and further purification for the production of rhOSM with isotope enrichment. Bioactivity of the rhOSM was comparable to commercially available recombinant human OSM (chOSM) in human breast cancer cells as determined by an enzyme-linked immunosorbent assay (ELISA), which detected phosphorylation of STAT3. Optimized buffer conditions for NMR studies were identified using differential scanning fluorimetry (DSF). The resulting NMR spectra showed an increase in the intensity of spectral peaks consistent with enhanced solubility of the rhOSM in conditions suitable for structure-based drug development. Moreover, we demonstrate the utility of the isotopically enriched rhOSM with ^1^H, ^15^N HSQC NMR titrations with, to the best of our knowledge, the first small molecule ligand determined to directly bind to OSM in binding site III. The results presented herein suggest that bioactive rhOSM can be efficiently produced and used for structure-based drug design.

## Results

### Expression and purification of rhOSM

The construct of rhOSM we have chosen to work with is a fragment of OSM encoding residues 1–187. This fragment was chosen for three reasons: (1) it has the same sequence used in the crystal structure and therefore supports a structure-based drug design approach^28^, (2) the binding site we are interrogating with small molecules is distal to the C-terminus, and (3) the C-terminus of the mature protein, comprising residues 188–196, does not contribute to OSMRβ binding^30^. In our initial efforts to express rhOSM, residues 1–187 of OSM were cloned into the plasmid pD441-NH (6-His-OSM), which has an amino terminal (N-terminal) tobacco etch virus (TEV) protease cleavage site (Fig. 1D). Expression of 6-His-OSM was tested in *E. coli* BL21(DE3) and BL21(DE3) pLysS cells grown in Lysogeny Broth (LB), minimal defined growth media (M9) or autoinduction medium. While cultures grew to high optical density (OD_600_ = 4.0–4.5) following induction with 1-thio-β-D-galactopyranoside (IPTG), the expression of 6-His-OSM was inconsistent and moderate at best. After examining a range of expression conditions (see Supplementary Information section “Expression optimization of 6-His-OSM” and Supplementary Figs. S1 & S2 for details) we hypothesized that the low yield of OSM was due to improper disulfide bond formation. Consequently, we tested OSM expression in Origami 2 pLysS and Shuffle cell lines, which are engineered with mutations in the thioredoxin and glutathione reductase genes and can enhance disulfide bond formation in cytoplasmic expression of recombinant proteins. Consistent expression was observed when 6-His-OSM was expressed in both Origami 2 pLysS and SHuffle cell lines (Supplementary Fig. S1). However, the Origami 2 pLysS consistently yielded a lower final cell density (data not shown). Further, the Origami 2 pLysS strain is auxotrophic and would have required isotope-enriched leucine supplementation for expression in minimal media, which would add a substantial cost to the protein production^34^. These disadvantages of the Origami 2 pLysS made the SHuffle cell line the optimal choice of OSM expression for NMR.

**Figure 2:**
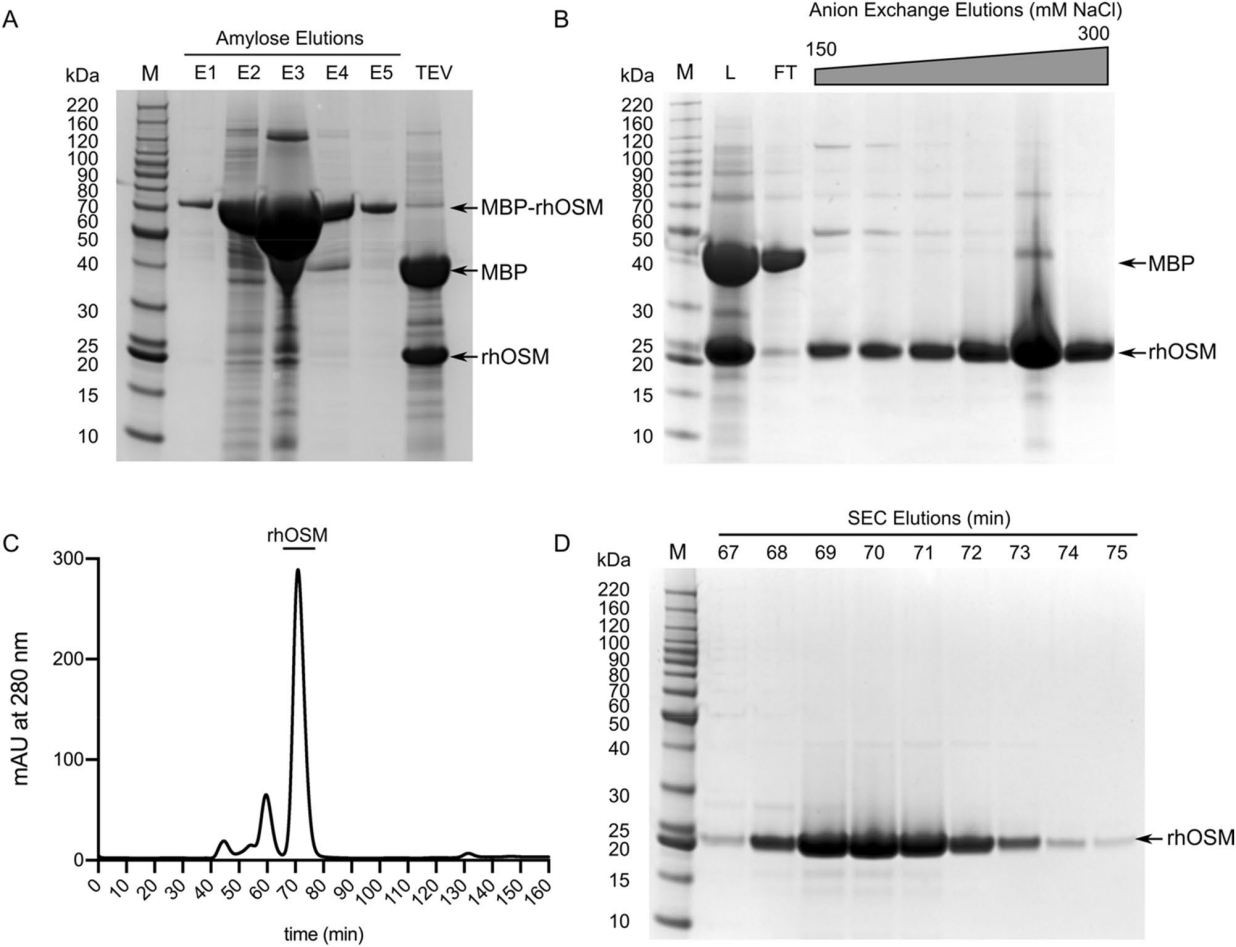
Expression and purification of rhOSM. (**A**) SDS-PAGE of amylose column purification of MBP-OSM, showing elutions E1-E5 and after cleavage with TEV. (**B**) SDS-PAGE of the load (L), flow-through (FT), and anion-exchange chromatography fractions. The fractions shown in gel were pooled and run over a size exclusion chromatography (SEC) column. (**C**) Chromatogram from SEC with rhOSM peak shown with bar. (**D**) SDS-PAGE of gel fractions from the SEC peak.

To increase solubility, OSM residues 1–187 were cloned into the pD441-MBP (MBP-OSM) expression construct, which contains an N-terminal TEV protease cleavage site. The combination of the SHuffle cell line and inclusion of the MBP solubility tag resulted in expression of increased soluble OSM, relative to the 6-His-OSM, as observed in SDS-PAGE (Supplementary Fig. S3). To further improve the yield of soluble OSM, we examined the effect of temperature on protein expression. Expression at 30 °C afforded higher yield of MBP-OSM relative to the expression at 18 °C (Supplementary Fig. S3) with no evidence of protein degradation even after a 20-h induction time. The final cell density was nearly twice as high with a 20-h induction relative to the 6-h induction. The expression of MBP-OSM with optimized conditions was scaled up to 1L growths in LB, ^15^N M9, and ^13^C, ^15^N M9. Three chromatography steps (amylose affinity, ion-exchange, and size-exclusion chromatography) were essential to prepare highly pure rhOSM (Fig. 2, Supplementary Figs. S5–S10). The purification procedure yielded 11.74 mg of rhOSM from 1 L of LB culture, 8.74 mg of ^15^N rhOSM from a 1L ^15^N M9 culture and 10.54 mg of ^13^C, ^15^N OSM from a 1L ^13^C, ^15^N M9 culture that was consistently > 99% pure as assessed by SDS-PAGE (Fig. 2D). Expression and purifications on a smaller scale (100 mL cultures) were performed in triplicate and showed consistency and reproducibility of the reported expression and purification of rhOSM, although lower yield (ca. 2–3 mg/L) was obtained from the 100 mL cultures (Supplementary Table 1).

### Biological activity of rhOSM

**Figure 3:**
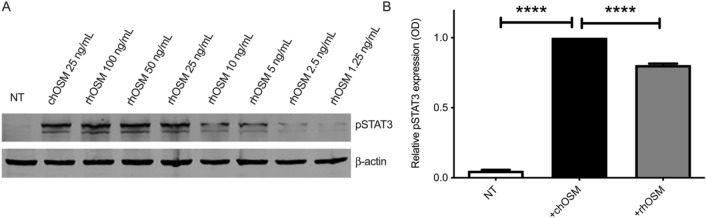
Recombinant OSM can effectively activate the STAT3 pathway in human breast cancer cell line, T47D. (**A**) T47D cells were untreated (NT), treated with commercial OSM (chOSM) or treated with increasing concentrations of recombinant OSM (rhOSM) for 30 min and levels of phosphorylated STAT3 (PSTAT3;Y705) were evaluated via immunoblot assay (representative blot from n = 2). pSTAT3 induction within cells treated with rhOSM show a similar trend to commercial OSM. Uncropped image shown in Supplemental Fig. S11. (**B**) Relative pSTAT3 expression in T47D breast cancer cells with no treatment (NT; white bar), or treatment with 25 ng/mL chOSM (black bar), or 25 ng/mL rhOSM (grey bar) over 30 min show significant increase in pSTAT3 levels detected via ELISA assay compared to NT and significance was observed between both treatment groups. Data expressed as mean ± SD from three replicates by one-way ANOVA with Tukey’s post-test (**** p value < 0.0001).

Extracellular OSM primarily stimulates the JAK/STAT3 pathway through interactions with OSMR^22^. To assess the biological activity of the rhOSM, phosphorylation at Tyr705 of STAT3 (pSTAT3) was measured in T47D human breast cancer cells using both immunoblot analysis as well as an enzyme-linked immunosorbent assay (ELISA). The pSTAT3 expression induced at concentrations ranging from 1.25 to 100 ng/mL rhOSM compared favorably with commercially available human OSM (chOSM), demonstrating similar efficacy at 25 ng/mL (Fig. 3A and Supplemental Fig. S11). As measured by ELISA, both rhOSM and chOSM induced significant pSTAT3 expression in T47D breast cancer cells compared to no treatment (Fig. 3B). Cells treated with chOSM displayed increased expression of pSTAT3 compared to rhOSM treated cells, the chOSM included additional 22 amino acids on the C-terminus that have been shown to preferentially bind to extracellular matrix molecules, potentially increasing the local concentration of the chOSM near the cell surface of the adherent T47D cells, leading to a predictable slight increase in pSTAT3 induction from chOSM relative to rhOSM. The additional C-terminal 22 amino acids are unstructured and are not in proximity to site III, thus were not included in the rhOSM construct.

### Secondary structure analysis by circular dichroism

**Figure 4:**
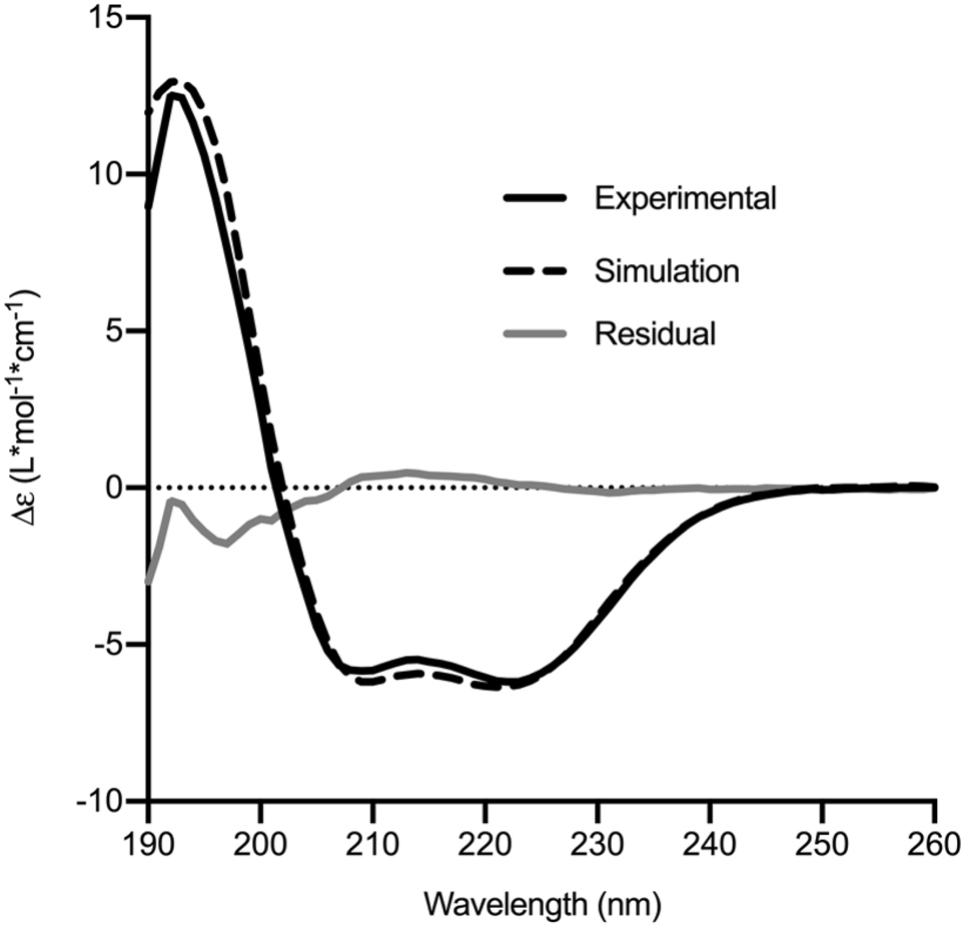
Circular dichroism spectrum shows rhOSM secondary structure is consistent with X-ray structure. An experimental spectrum of rhOSM (solid, black) is compared to a simulated spectrum (dashed, black) derived from a model of the X-ray crystal structure with the flexible Loop AB that was missing electron density in the crystal structure modeled in. Residuals between the experimental and simulation data (grey) are low at higher wavelengths but increase at lower wavelengths due to solvent scattering.

Circular dichroism (CD) spectroscopy can be used to assess the secondary structure of proteins in solution and is useful as a quality control step in protein production^35^. A CD spectrum of rhOSM showed that the purified protein was mostly alpha-helical with secondary structure contributions consisting of 56% helix, 8% sheet, 11% turn, and 26% unordered. This result was compared with a CD spectrum simulated using a structural model of human OSM generated from the crystal structure^28^, which modeled in parts of the protein that were missing electron density (Fig. 4). The predicted secondary structure contributions from the structural model were 61% helix, 0% sheet, 10% turn, and 28% unordered with an RMSD of the experimental to the simulated spectrum of 0.66. The experimental and simulated spectra are in close agreement, suggesting the rhOSM has a secondary structure closely resembling the OSM crystal structure.

### Mass spectrometry analysis of rhOSM

The sequence and disulfide pattern of rhOSM was analyzed by mass spectrometry. Purified rhOSM was subjected to tryptic digestion in the presence and absence of 2-iodoacetamide (IAA). Alkylation of free cysteine thiols with IAA results in an S-acetamide derivative that adds 57 mass units^36^, which allows for detection of free thiols by comparing the untreated sample with the alkylated sample. The resulting peptide fragments were detected by MALDI-TOF mass spectrometry, and mass-to-charge (*m/z*) values of the resulting peptide ions were analyzed. The sequence coverage was 96% in the absence and 91% in the presence of IAA (Supplementary Fig. S12). The highest intensity peak in the absence of IAA (1743.0 m*/z*) was identified as the peptide 69-GFLQTLNATLGCVLHR-84 (Supplementary Fig. S13 and Table S2). In the IAA treated sample, the highest intensity peak shifted to 1799.9 m*/z* (Supplementary Fig. S14 and Table S3), consistent with the expected + 57.0 Da mass shift with the addition of the S-alkylated acetamide derivative at Cys80. A confirmative set of peaks at 1899.1 m*/z* and 1965.1 m*/z* were identified as the unmodified and an S-alkylated acetamide derivative at Cys80 of fragment 68-RGFLQTLNATLGCVLHR-84. Several peaks heavier than 2200 m*/z* were observed in the untreated sample and could be assigned to Cys6-Cys127 and Cys49-Cys167 disulfides but could not be discriminated from combinations of peptides that also could have contained the Cys80 residue. All of the expected disulfide peptides (> 2000 m*/z*) were low intensity in the untreated sample, and none were observed in the IAA treated sample (maximum detected peak was 2480.3 m*/z*). This suggests that the higher *m/z* peaks were due to interaction between cysteine containing peptides and the free Cys80 during ionization and were not necessarily representative of interactions in solution. A similar observation of low intensity or missing peptides that should have indicated proper disulfide formation in OSM has been reported before^37^.

### Optimization of buffer conditions for structural studies

**Figure 5:**
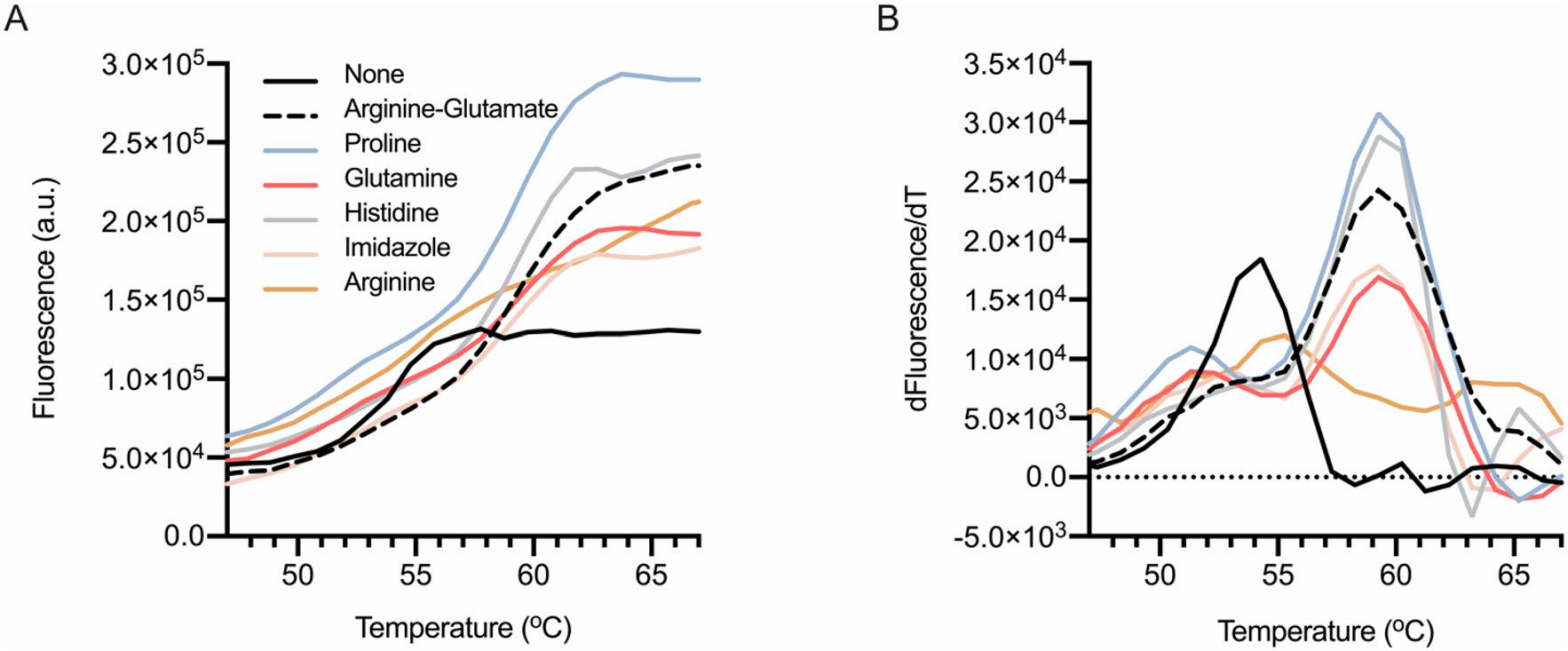
Additives increase the thermal stability of rhOSM in pH 6.6 phosphate buffer. (**A**) Thermal denaturation curves with legend. Curve with sodium phosphate buffer only (None), shown in black; L-arginine and L-glutamate in dashed black; L-proline in sage; L-glutamine in salmon; L-histidine in gray; imidazole in peach; and L-arginine in orange. (**B**) The first derivative of fluorescence emission as a function of temperature.

In vitro solution studies of rhOSM, including NMR, require a protein to behave as a homogeneous, stable solute. Specifically, for NMR experiments, a phosphate buffered system is preferred over protonated buffer components and additives, which potentially distort the spectra, making interpretation more challenging. We used differential scanning fluorimetry to screen additives in a phosphate buffer that would increase the thermal stability of rhOSM. Various amino acid and polyamine additives were investigated using an established approach^38^. The denaturation profile of rhOSM in sodium phosphate buffer with and without additives exhibited low fluorescence at room temperature, indicative of a well-folded protein (Fig. 5A). All additives except L-arginine showed a noticeable stabilization of rhOSM. Imidazole, L-histidine, L-glutamine, L-proline, and a combination of L-arginine and L-glutamate equally increased melting temperature from 54 ºC to 59 ºC (Fig. 5B and Table 1). The L-arginine and L-glutamate combination is known to prevent protein aggregation and precipitation, increasing long-term stability, and protecting protein samples from proteolytic degradation^39^. We observed a decrease in proteolysis of rhOSM with L-arginine and L-glutamate relative to no additive (Supplementary Fig. S15). Moreover, it has been demonstrated that the presence of these amino acids does not interfere with protein–protein interactions^39^, thus we chose to use the L-arginine and L-glutamate combination in NMR experiments.

**Table 1:** Summary of the melting temperatures for the largest amplitude peak from the sum of two Gaussians fit to the first derivative of fluorescence with respect to temperature for each of the additive conditions.

Additive	Concentration (mM)	T_*m*_ (°C)
None	0	54.3
Arginine-Glutamate	50–50	59.4
Proline	100	59.4
Glutamine	100	59.5
Histidine	15	59.4
Imidazole	5	59.3
Arginine	50	54.3

**Figure 6:**
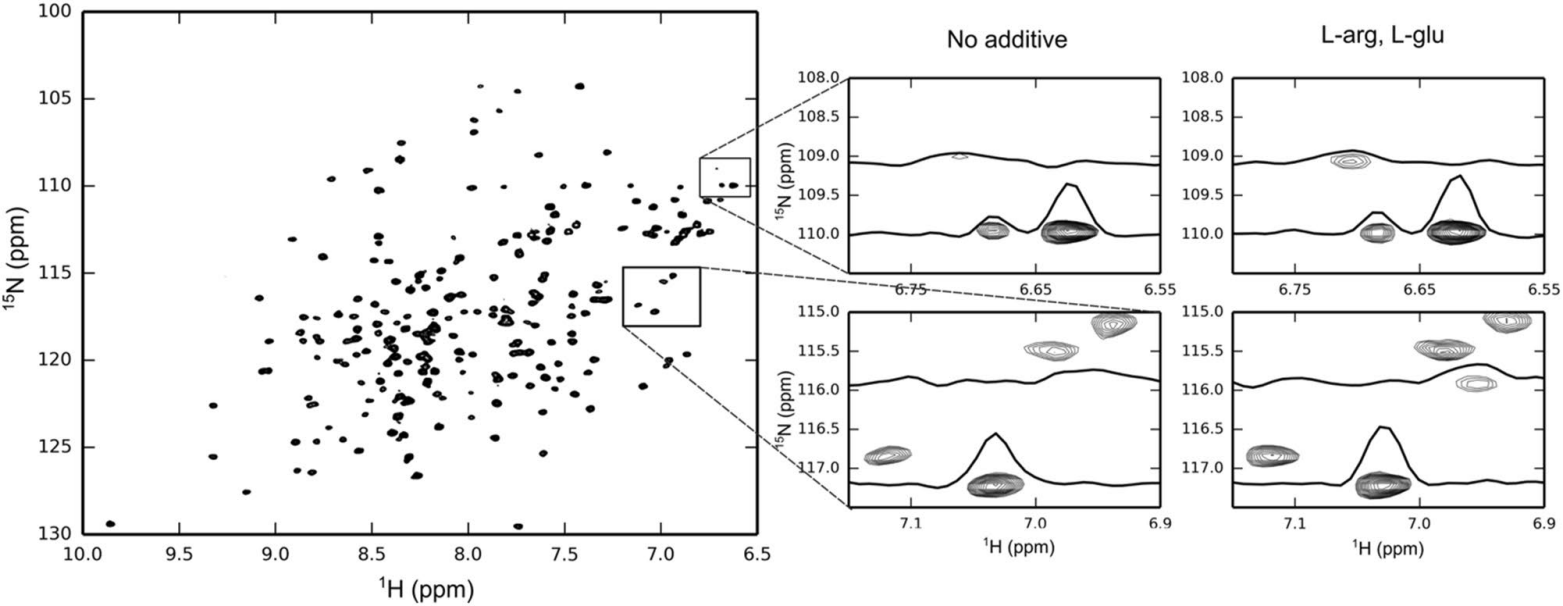
Optimized buffer conditions improve the quality OSM NMR spectrum. ^1^H, ^15^N HSQC spectrum of 100 μM ^15^N-labeled OSM in 50 mM sodium phosphate, 100 mM sodium chloride, pH 6.6. Zoomed regions of interest from the ^1^H,^15^N HSQC spectrum and projection along the ^15^N axis of 100 μM ^15^N-labeled OSM with no additive (left) and with 50 mM L-arginine, 50 mM L-glutamate (right). Spectra were collected with identical parameters, processed the same and plotted at the same contour levels.

To assess the effect of the additives on the structural stability of rhOSM, a ^1^H, ^15^N heteronuclear single quantum correlation (HSQC) NMR spectroscopy was collected. Spectra were recorded in a 50 mM sodium phosphate buffer, pH 6.6; without and with 50 mM L-arginine and 50 mM L-glutamate additive (Fig. 6). Both spectra show dispersed, resolved peaks, indicative of a well-folded protein, however, the optimized buffer conditions using 50 mM L-arginine and 50 mM glutamate displayed increased signal to noise ratio across the spectrum (Fig. 6). The addition of L-arginine and L-glutamate allowed for detection of several peaks that were not observable in the absence of the additive (Fig. 6). Overall, the spectral quality was increased in the presence of L-arginine and L-glutamate.

### Small molecule inhibitor titrations

After identifying optimal solution conditions for ^1^H, ^15^N HSQC NMR experiments with ^15^N rhOSM, we focused our efforts toward assessing the utility of this construct in a structure-based drug design approach to target OSM. Upon titration of an isotopically enriched protein with a ligand of sufficient binding affinity, changes in the local magnetic environment of backbone amide bonds (N–H) give rise to characteristic chemical shift perturbations (CSPs)^40^. If backbone N–H correlation resonances are assigned, this assay allows for identification of interacting residues at the small molecule/protein interface and assignment of putative binding modes. Further, nonlinear regression analysis of the magnitude of the CSPs plotted against concentration of small molecule delivered may be used to determine a binding affinity measured as the dissociation equilibrium constant (*K*_*d*_) for the small molecule inhibitor (SMI).

**Figure 7:**
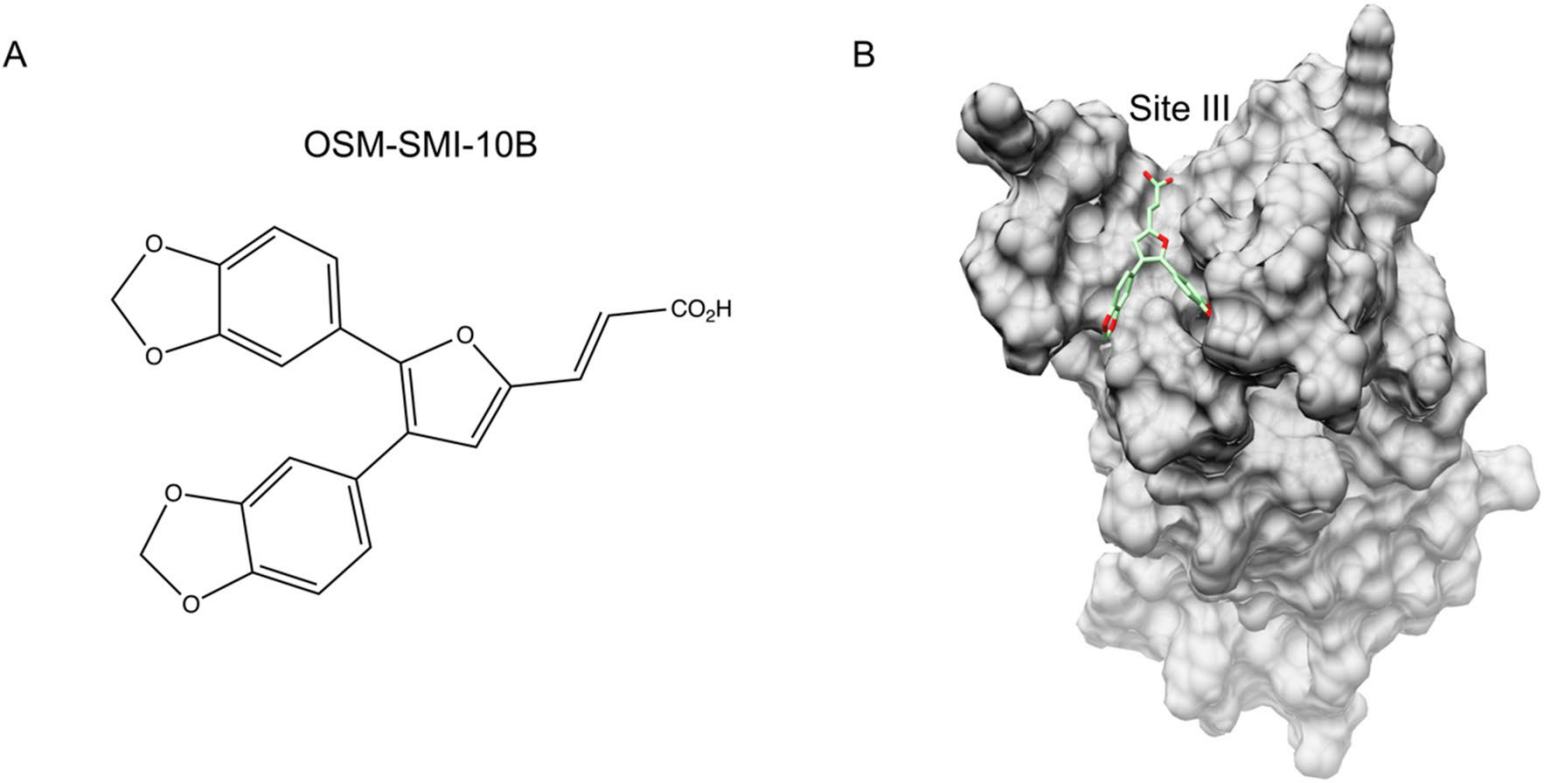
OSM-SMI-10B docked to OSM. (**A**) Chemical structure of OSM-SMI-10B. (**B**) OSM-SMI-10B (green and red) docked into a groove found at Site III on OSM (surface contour, grey).

Here, we chose to test the NMR assay using rhOSM and a preliminary inhibitor identified from a previous screen (unpublished). Briefly, a small molecule inhibitor site was identified that was expected to directly block protein–protein interactions with the OSMRβ subunit at site III. A virtual library consisting of ~ 1.6 million compounds^41,42^ was screened and prioritized based on their predicted binding energy potential to binding sites (unpublished). The top 26 candidates were purchased and tested in vitro for inhibition of OSM-induced phosphorylation of STAT3 (unpublished). Lead compound OSM-SMI-10 was identified as the most potent inhibitor of pSTAT3^43^. OSM-SMI-10B (Fig. 7A,), a derivative of OSM-SMI-10 (patent pending) was subsequently synthesized and identified as a hit based on its ability to significantly reduce OSM-induced STAT3 phosphorylation in cancer cells upon co-incubation with OSM as measured by ELISA^44^. Supplementary Information section “Chemistry” provides details regarding the synthesis of OSM-SMI-10B. Characterization of intermediates and final product are shown in Supplemental Scheme 1 and Figs. S16–S2. OSM-SMI-10B was chosen for this study to demonstrate the utility of an NMR based screen to optimize the structure-based drug design approach to inhibit OSM.

**Figure 8:**
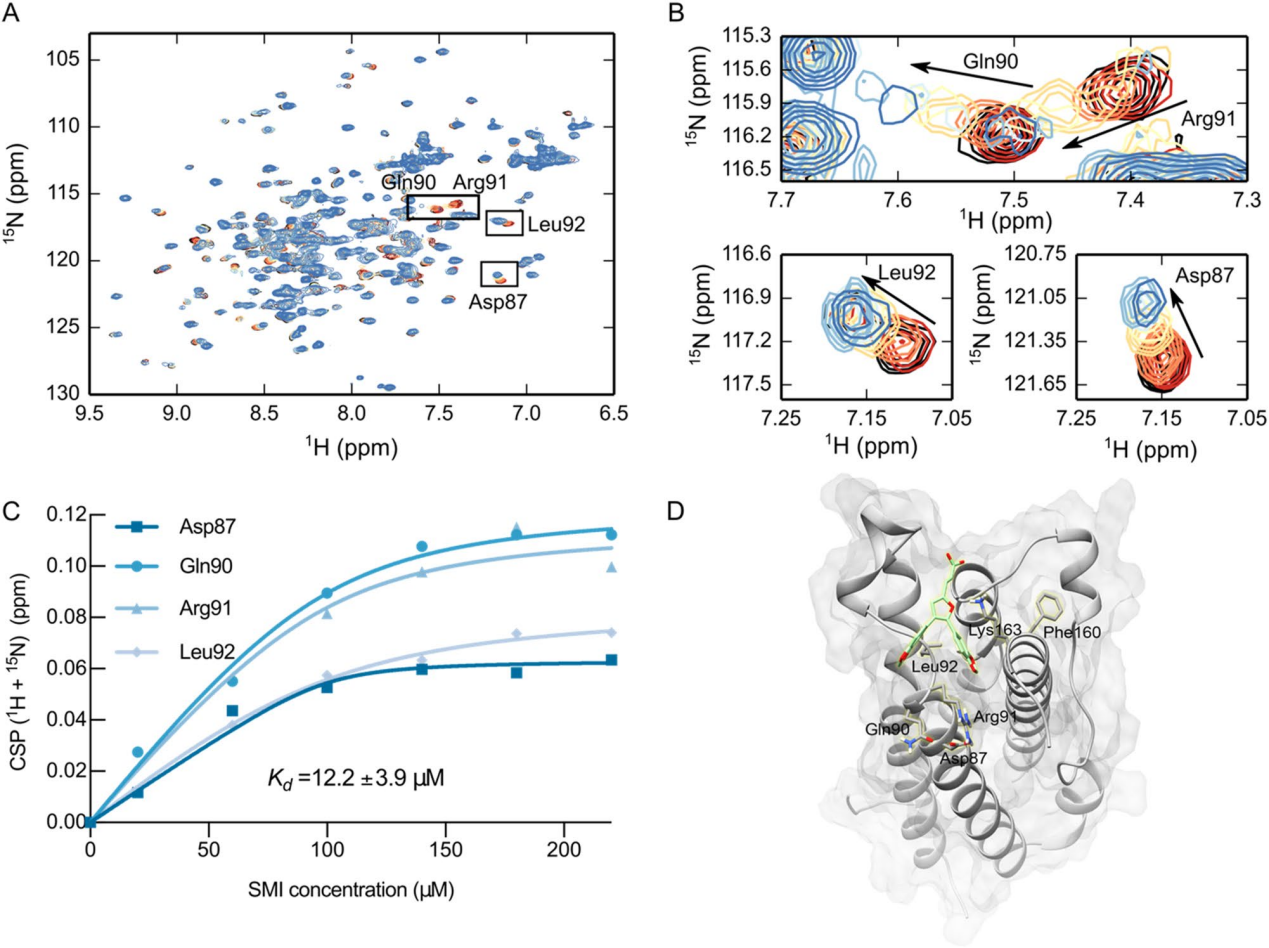
SMI titration of ^15^N rhOSM monitored by NMR spectroscopy. (**A**) An overlay of ^1^H, ^15^N HSQC NMR spectra of 100 μM ^15^N-labeled OSM with cross peaks color-ramped from red to blue with increasing SMI concentration from 20 μM (red) to a final concentration of 220 μM (blue). A DMSO control is shown in black. (**B**) Zoomed regions of panel A for Asp87, Gln90, Arg91, and Leu92. (**C**) A saturation binding curve was fit to the CSP vs. SMI for peaks that were tentatively assigned to Asp87, Gln90, Arg91, and Leu92. (**D**) OSM oriented with Site III in view with protruding Phe160 and Lys163 residues (shown as sticks and colored gold), which make up an FXXK motif in binding site. The side chains of residues Asp87, Gln90, Arg91, and Leu92, which show significant CSPs are shown as sticks and labeled.

OSM-SMI-10B was computationally docked into Site III (Fig. 7B), which is considered the interaction site for OSMRβ. To determine if the decrease in OSM activity was the result of direct binding to OSM and to identify the OSM-SMI-10B binding site, ^15^N rhOSM was titrated with increasing concentration of OSM-SMI-10B (Fig. 8A–C). Significant CSPs were observed for multiple peaks. The four peaks with the largest CSPs were selected and analyzed using the quadratic expansion of the isotherm binding model to obtain a *K*_*d*_ of 12.2 ± 3.9 μM. Given the experimental conditions, the calculated *K*_*d*_ is an estimate of an upper limit of the binding affinity. The peaks used for fitting were tentatively assigned to Asp87, Gln90, Arg91, and Leu92 by comparing the minimal chemical shift differences between rhOSM and a glycosylated version of hOSM for which peak assignments available^45^. Additional assignments were not possible due to differences in primary sequence and post-translational modifications. Notably, Asp87, Gln90, Arg91, and Leu92 are all located near OSM binding site III (Fig. 8D), where OSM is thought to interact with the OSMRβ subunit^28^. The *K*_*d*_ was further validated with a fluorescence quenching assay, which yielded a *K*_*d*_ of 10.37 ± 0.81 μM (Supplemental Figs. S24 & S25). A comparison of the NMR and fluorescence quenching experiments to two additional SMI compounds is shown in Supplementary Figure S25A. OSM-SMI-10B11 is a derivative of OSM-SMI-10B and OSM-SMI-27A6 is chemically unique SMI, synthesized based on QSAR and computational modelling. The binding affinities were similar between OSM-SMI-10B and OSM-SMI-10B11 and the corresponding CSPs had a similar pattern (Supplementary Figure S25C). OSM-SMI-27A6 was twofold weaker in affinity and showed fewer CSPs common to the other two SMIs. However, due to insolubility, the maximum titration point for OSM-SMI-27A6 was only 80 μM Supplementary Figure S25D. This comparison highlights the utility of NMR to validate direct binding of SMIs to target proteins.

## Discussion

The role of OSM in multiple inflammatory diseases including fibrotic diseases, arthritis, and numerous types of cancers has led to significant interest in attenuating OSM cell signaling as a potential therapeutic. Despite advances in biological therapies such as monoclonal antibodies to inhibit OSM, there are no FDA-approved anti-OSM drugs. Moreover, there have been no reports of small molecules that specifically bind to and inhibit OSM. This is presumably due to the fact that OSM exerts its biological activity through protein–protein interactions (PPIs), which are notoriously difficult to disrupt with small molecules. PPIs are generally characterized by large, flat, discontinuous, and hydrophobic binding interfaces that are not well-suited for high specificity and affinity small molecule binding^46^. Though targeting PPIs has been a major challenge in drug discovery, structure-based NMR experiments have become essential to targeting these interactions that were once thought to be “undruggable”^47^. For example, using NMR screening and structure-based drug design, highly potent small molecule inhibitors of Bcl-2 family protein–protein interactions were discovered^33^ that eventually led to the development of FDA-approved Bcl-2 inhibitor ABT-199 (Venetoclax)^48^. This serves as an important proof-of-concept that PPIs can be targeted using NMR-based approaches, and such inhibitors are amenable to clinical development. Further, interactions between Interleukin-2 (IL-2)/Interleukin-2 receptor alpha subunit (IL-2Rα) and their disruption by small molecules were previously studied using NMR techniques^49^. These structure-based methods require soluble, functional proteins at high yield, and as such this effort is essential to pursuing structure-based drug discovery efforts for targeting OSM.

There are reports of recombinant expression of human OSM, but previous methods had either low yields, low purity, or inhomogeneous protein. For example, a 6-His tagged OSM was expressed in *E. coli* BL21(DE3) but required refolding in the presence of glutathione reductase to facilitate proper disulfide bond formation. Though this protocol allowed for a one-step purification using immobilized metal affinity chromatography, OSM was obtained in relatively low yield (2 mg/L culture)^50^. Soluble recombinant OSM was produced via a GST-fusion in the *E. coli* JM109 strain, though purification details and yield were not included^51^. Recombinant OSM was similarly expressed for crystal structure determination via GST-fusion in *E. coli* JM109, consisting of residues 1–187 with four extra residues (GPGS) corresponding to a 3C protease cleavage site. The OSM was reportedly greater than 80% pure as determined by SDS-PAGE after purification, but again yields were not included^28^. Isotope enriched OSM was previously produced for solution NMR studies. However, this protocol involved expression from yeast *Saccharomyces cerevisiae,* C80A/N30A/N192A mutations, a non-native FLAG (FYDDDDK) affinity tag appended to the N-terminus, and post-translational modifications that resulted in a heterogeneous mixture; together complicating resonance assignment^45^. Though the mutated OSM construct was shown to have similar activity to radiolabeled native OSM in a competitive LIFR binding assay, it is unclear if the mutations have deleterious effects on the ability of OSM to bind to the OSMR and thus activate OSM-specific cell signaling pathway, as this was untested.

Here, we report successful production of recombinant unlabeled, ^15^N labeled, and ^13^C, ^15^N labeled rhOSM in milligram quantities with excellent purity (≥ 99%). The identity, purity and bioactivity of the rhOSM was fully characterized and the rhOSM concentration-dependent induction of pSTAT3 was similar to chOSM, as measured by immunoblot and enzyme-linked immunosorbent assays. Optimized buffer conditions enabled collection NMR spectra from both ^15^N and ^13^C, ^15^N labeled rhOSM samples with dispersed and well-resolved peaks, which indicate that the rhOSM is properly folded. Importantly, inhibition of OSM-driven pSTAT3 production by a small molecule and NMR titrations lay the groundwork for an NMR-based drug design approach to targeting OSM. Our protocol is similar to Nguyen et al., who used a maltose binding protein (MBP) fusion construct and expression in *E. coli* SHuffle^37^, which we independently also found. A notable distinction in our study is the bioactivity test. Nguyen et al. tested their recombinant human OSM for activity by in vitro inhibition of Th17 *mouse* cell differentiation. Critically, *human OSM* shares only 48.9% sequence identity with *mouse OSM* (mOSM) where differences within the AB loop of OSM determine species-specific activities^31^. Although hOSM is able to activate mouse LIFR (mLIFRα/gp130), hOSM and mOSM display *no cross-species* signaling to OSMR despite their structural similarities^22,52^. We used the T47D human breast cancer cell line for the bioactivity assay, which we feel is a more appropriate cell line for small molecule lead optimization screening.

Reports of small molecules with anti-OSM activity are scarce, both in the journal and patent literature^43^. Members of the ansamycin family of antibiotic microbial secondary metabolites were shown to potently inhibit OSM and IL-6 driven secreted placental alkaline phosphatase (sPAP) production in HepG2B6 cells while having little to no effect on TNFα sPAP production. However, the ability of the same compounds to inhibit protein synthesis and lymphocyte production suggested their biological activities were non-specific and were unlikely to be attributable to direct inhibition of OSM or IL-6^53^. N-(1H-pyrazolo[3,4-d]pyrimidin-4-yl)benzamide was similarly shown to suppress OSM driven sPAP production in HepG2B6 cells with limited inhibition of TNFα driven sPAP production, but it is unclear if this activity is attributable to specific interaction with OSM, as this was otherwise untested^54^. Though these studies potentially identified promising lead compounds for attenuating OSM signaling, the methods employed were unsatisfactory for inhibition was due to direct interaction of small molecules with OSM. This issue is compounded by the high structural similarity of OSM with other cytokines in its family, namely IL-6 and LIF. The development of anti-OSM therapeutics without careful analysis of specific OSM/SMI interactions would likely have significant cross-reactivity with these cytokines. In an effort to alleviate these potential issues, we sought to develop a reliable methodology for directly measuring OSM/SMI interactions.

NMR-based experiments are important tools for drug discovery and design and have been used to elucidate structure–activity relationships and develop high-affinity small molecule ligands^32^, fragment-based screening and drug design^55^, high-throughput screening and determination of binding affinity^56^, and mapping important binding interactions of target proteins^40^ and small molecule ligands^57^. Here, we utilized ^1^H, ^15^N NMR chemical shift perturbation experiments to confirm direct binding of a small molecule inhibitor of OSM-driven phosphorylation of STAT3. Several peaks in the ^1^H, ^15^N NMR spectrum with chemical shift perturbations were tentatively assigned to residues in binding site III of OSM, where OSM interacts with the OSM-specific OSMRβ. This is the first report of a small molecule confirmed to modulate OSM activity through direct binding to OSM. These findings suggest that small molecules can be used to specifically target the OSM-OSMRβ interaction with biologically relevant affinities (*K*_*d*_ ≈ 10 μM). The isotopically enriched rhOSM produced using our methodology will enable NMR-based experiments for further characterization of OSM-small molecule interactions that will inform iterative optimization of the SMIs. Current efforts are underway in our lab to obtain full backbone and side chain chemical shift assignments for rhOSM. We envision that using our optimized bioactivity assay in combination with NMR chemical shift perturbations will enable a structure-based drug design approach for targeting OSM that could eventually lead to a useful therapeutic option for treatment of OSM-related illnesses.

## Methods

### Reagents

Unless otherwise mentioned, chemical reagents were purchased from Fisher Scientific (Waltham, MA). Isotopically enriched reagents were purchased from MilliPoreSigma (St Louis, MO).

### Plasmid constructs

6-His-OSM and MBP-OSM constructs were purchased from ATUM (Newark, CA), amplified in DH5α *E. coli* chemically competent cells and purified using a PureLink Quick Plasmid Miniprep kit; both from Invitrogen (Carlsbad, CA).

### Recombinant OSM expression

Freshly transformed chemically competent Shuffle *E. coli* cells were used to inoculate 10 mL LB containing appropriate antibiotics at 30 °C, overnight, shaking at 220 rpm. The overnight culture was used to inoculate 1 L cultures appropriate antibiotics at starting OD_600_ = 0.05–0.10. Media was either LB, M9 supplemented with (≥ 98 atom %) ^15^N ammonium chloride and 0.5 g/L ^15^N Isogro, or (≥ 98 atom %) ^15^N ammonium chloride, (≥ 99 atom %) ^13^C glucose and 0.5 g/L ^15^N, ^13^C Isogro). The low level of labeled algal lysate (Isogro) is included to decrease the time to induction (see Supplementary Fig. S4). The cells were grown at 37 °C with shaking to OD_600_ = 0.6–0.8. Expression was induced with 1 mM IPTG and cultures were grown for 20 h at 30 °C with shaking. Cells were harvested by centrifugation, suspended in Lysis Buffer and stored at − 20 °C until purification.

### Purification of recombinant OSM

Frozen re-suspended cells were thawed and immediately disrupted by sonication in an ice-salt bath. The lysate was clarified by centrifugation and filtered through a 0.4 micron syringe-driven filter. The cleared lysate was loaded onto a pre-equilibrated amylose resin (New England BioLabs, Ipswich, MA), and the flow through was collected. The column was washed with Binding Buffer (20 mM Tris–Cl, 200 mM sodium chloride, 1 mM EDTA, 1 mM DTT, pH 7.4), then eluted with a linear gradient of elution buffer (Binding Buffer with 10 mM maltose). Eluted MBP-OSM was cleaved with TEV protease (1 mg of TEV protease per 100 mg of MBP-OSM) overnight at room temperature in 50 mM sodium phosphate, 100 mM sodium chloride, 0.5 mM EDTA, 1 mM DTT, pH 7.5. TEV protease is prepared in-house using a standard protocol. The resulting solution was then dialyzed into a lower salt buffer (50 mM sodium phosphate, 10 mM sodium chloride, pH 7.5) and applied to Macro-prep High S resin (Bio-Rad, Hercules, CA) and eluted with a linear sodium chloride gradient to remove cleaved MBP and TEV. Fractions containing OSM were identified by SDS-PAGE, combined, and concentrated. The sample was filtered using a 10 μm spin column and subjected to size-exclusion chromatography (Superdex S75 16/60; Cytiva, Marlborough, MA) in a buffer containing 50 mM sodium phosphate, 100 mM sodium chloride, 1 mM DTT, pH 6.6. Fractions containing OSM were identified from the chromatogram and confirmed by SDS-PAGE. The concentration of purified OSM was determined spectroscopically at 280 nm using the calculated extinction coefficient of 11,460 M^−1^⋅cm^−1^.

### SDS-PAGE

Aliquots of pre-induced and post-induced cells were taken in an amount corresponding to OD_600_ = 0.8 in 1 mL, pelleted and boiled in 100 μL SDS-PAGE Sample Buffer containing 10% (v/v) 2-mercaptoethanol prior to gel electrophoresis. Aliquots from chromatography samples were diluted fourfold in Sample Buffer and boiled prior to gel electrophoresis. Samples were run on NuPAGE 4–12% Bis–Tris gels (ThermoFisher Scientific, Waltham, MA) and stained with SimplyBlue SafeStain (ThermoFisher Scientific, Waltham, MA). Gel images were collected with a FluorChem R imager (ProteinSimple, Santa Clara, CA). Protein expression and percent purity were quantified from gel images using ImageJ (http://imagej.nih.gov/ij).

### Bioactivity assays

#### Cell lines

The human T47D breast cancer cell line was purchased from the American Type Culture Collection (Rockville, MD, USA) and cultured using RPMI 1640 media supplemented with 10% Fetal Clone III synthetic serum (HyClone Cytiva, Marlborough, MA; Cat# SH30109.3) and 100 units/mL penicillin/streptomycin. All cells and experimental incubations were maintained at 37 °C, 5% carbon dioxide, and 100% humidity within a water-jacketed cell culture incubator.

#### Immunoblot analysis

T47D cells were plated on 24-well plates at 70–75% confluency and allowed to adhere overnight. Cells were serum starved with serum-free RPMI media for 4 h and subsequently treated with either commercially available recombinant human OSM (chOSM) (PeproTech, Rocky Hill, NJ; Cat# 300-10 T) or rhOSM at specified concentrations for 30 min. Afterwards, cell lysates were collected with 1X Cell Lysis Buffer (Cell Signaling Technology, Danvers, MA; Cat# 7018). Lysates were run on an SDS-PAGE gel and transferred onto a nitrocellulose membrane via semi-dry transfer. Blots were rinsed in ddH_2_O and allowed to dry completely before being blocked with LiCor Odyssey PBS blocking buffer (LiCor, Lincoln, NE; Cat# 927-4000) for 1 h. After blocking, primary antibodies (1:1000) suspended in blocking buffer were applied to the membrane, shaken for 1 h at room temperature, and incubated overnight at 4 °C. Membranes were then washed 6 × 5 min with 1X PBS supplemented with 0.5% Tween and secondary antibodies (1:15,000) suspended in blocking buffer were applied for 45 min. A final wash step of 6 × 5 min with PBS-T, membranes were imaged at 700 nm using the LiCor Odyssey CLx imaging system. Antibodies: phospho-STAT3 (Y705) (Cell Signaling Technology, Danvers, MA; Cat# 9145), beta-Actin (Cell Signaling Technologies; Cat# 3700), donkey anti-rabbit IRDye 800CW (LiCor, Lincoln, NE; Cat# 925-32213).

#### Enzyme linked immunosorbent assay (ELISA)

T47D cells were plated as on 24-well plates at 70–87% confluency and allowed to adhere overnight at 37 °C. Following overnight incubation, cells were serum starved with serum free RPMI media for 4 h and subsequently treated with 25 ng/mL chOSM (PeproTech, Rocky Hill, NJ; Cat# 300-10 T) or rhOSM at 25 ng/mL for 30 min. Afterwards, lysates were collected as previously described for immunoblot^20^. Lysate samples were diluted 1:4 in 1X PBS supplemented with 1% BSA. Levels of phospho-STAT3 were detected using the PathScan Phospho-STAT3 (Y705) Sandwich ELISA kit (Cell Signaling Technologies; Cat# 7300) as per manufacturer's instructions. Relative expression was quantified comparing non-treated and rhOSM treated samples to chOSM levels. Experiments were run in triplicate and data was analyzed using Microsoft Excel and Prism 8 (v. 8.2.1, GraphPad Software).

### Circular dichroism

Purified rhOSM was exchanged into 50 mM sodium phosphate buffer, pH 6.6, containing 100 mM NaF, and 1 mM TCEP on an S75 column. CD spectra were collected using a Jasco J-810 spectropolarimeter (JASCO). Purified rhOSM was measured at 0.197 mg/mL in a 0.1 cm cuvette at 25 °C for far-UV scans (275–170 nm). The background subtracted CD spectrum of rhOSM and $\Delta \varepsilon$ was calculated using the equation:

$$\Delta \varepsilon =\theta \times \frac{0.1\times MRW}{P\times CONC\times 3298}$$

where θ = machine unit output, mean residue weight (MRW) = protein mean weight (daltons)/number of residues, path length (P) = cuvette path length in cm, protein concentration (CONC) = protein concentration in mg/mL, and [θ] = 3298 Δε. The resulting spectrum was then analyzed using the DichroWEB server^58^. The CDSSTR method with SP175 reference dataset were used to calculate the secondary structure contributions to the CD spectrum^59^ A structural model of OSM was generated using Swiss-MODEL^60^ to account for residues in the AB Loop that were missing from the crystal structure (1EVS^28^). A CD spectrum was simulated using the coordinates of the resulting model with the software PDB2CD^61^.

### Mass spectrometry

Purified rhOSM (30 μg) was diluted in Tris buffer (50 mM, pH 8.0), followed by the treatment with 1,4-dithiothreitol (DTT, 10 mM) to reduce disulfide bonds and iodoacetamide (IAA, 55 mM) for alkylation and then digested with trypsin (trypsin to protein ratio 1:30) at 37 °C overnight. In a separate experiment, purified rhOSM (30 μg) was digested directly with trypsin under the same conditions but without the treatment with DTT and IAA. Mixtures of resulting peptides was desalted using a Pierce C18 spin column (ThermoFisher Scientific, Waltham, MA), dried in a vacuum centrifuge, and reconstituted in water containing 0.1% trifluoroacetic acid (TFA). One μL of the peptide solution was mixed with one μL of saturated α-Cyano-4-hydroxycinnamic acid solution in 50% acetonitrile and 0.1% TFA. One μL of the mixture was then applied to a ground stainless steel MALDI target and allowed to air dry. Spectra of the peptides were acquired on a Bruker autoflex MALDI TOF mass spectrometer in reflector mode. The masses were analyzed using Protein Prospector v 5.24.0 (http://prospector.ucsf.edu/prospector/mshome.htm). Data were compared to theoretical masses from PeptideMass^62^ with allowance of one missed Trypsin cleavage.

### Differential scanning fluorimetry

rhOSM (560 μL, 33 μM) was incubated with 1 μL of SYPRO Orange, 5000X in DMSO (Invitrogen), thoroughly mixed and divided between eight vials. Concentrated stock solution of each additive was diluted to the final concentrations shown in Table 1. Each solution was loaded 3 × 20 μL into a 96-well clear reaction PCR microplate. The microplate was sealed with an adhesive optical clear seal and centrifuged at 500 × *g* for 2 min at room temperature. Data were collected on a QuantStudio3 qPCR system (Applied Biosystems). Samples were equilibrated at 25 °C for 5 min before starting a temperature ramp of 1 °C/min over a temperature range from 25 °C to 95 °C. Fluorescence readings were recorded every 1 min. According to the described protocol^38^ raw data were truncated in Excel to remove post-peak quenching, then analyzed and fit using a nonlinear Gauss equation in Prism 8 (v. 8.2.1, GraphPad Software). The thermal denaturation temperatures (T_*m*_) of rhOSM screened with buffer additives were determined by fitting a sum of two Gaussians to the time derivative of fluorescence. The T_*m*_ for each buffer condition is reported for the Gaussian fit with the maximum amplitude.

### Molecular docking

Molecular docking was performed using AutoDock Vina^63^. The human OSM protein structure (PDB ID: 1EVS) was used as the “receptor” model. The OSM structure was minimized using sequential steepest descent and conjugate gradient algorithms. OSM-SMI-10B was docked against OSM at Site III using a rigid receptor model, grid point spacing of 0.375 A, and exhaustiveness of 8. Predicted binding affinities were calculated using the default weighting for the AutoDock Vina scoring function.

### NMR spectroscopy

All NMR experiments were conducted in 50 mM sodium phosphate (pH 6.6), 100 mM sodium chloride, 5% D_2_O (NMR buffer) with 500 μL of sample in a 5 mm NMR tube unless otherwise noted. Spectra were recorded at 298 K on a Bruker AVANCE III 600 MHz spectrometer equipped with a 5 mm TCI cryoprobe with z-axis gradients. ^1^H, ^15^N HSQC spectra were collected with the Bruker pulse program *hsqcfpf3gpphwg*^64^. Spectra were processed with Topspin 3.2 (Bruker, Billerica, MA), NMRPipe^65^ and plotted using NMRglue^66^. For buffer optimization studies, HSQC spectra were taken of 100 μM ^15^N OSM in NMR buffer and in NMR buffer supplemented with 50 mM L-arginine, 50 mM L-glutamate. Samples were filtered with 10-micron spin filter prior to loading into NMR tube.

For small molecule inhibitor (SMI) titrations, 500 μL of a 100 μM sample of ^15^N OSM in NMR Buffer was titrated with a 20 mM stock solution of OSM-SMI-10B in d_6_-dimethyl sulfoxide. The SMI stock solution was added in 1 μL increments from 20 μM to a final concentration of 220 μM total OSM-SMI-10B. An additional 100 μM sample of ^15^N OSM was prepared with 8 μL d_6_-dimethyl sulfoxide as a control. The four peaks with the largest chemical shift perturbations (CSPs) were subjected to binding analysis using the difference in chemical shift of both ^15^N and ^1^H^40^. Combined CSPs for the four selected peaks were then plotted against the concentration of SMI delivered and fit to a saturation binding model, given by:

$${\Delta }_{\delta }= \frac{Kd+\left[L\right]+\left[P\right]-\sqrt{{(Kd+\left[L\right]+\left[P\right])}^{2}-4 \left[L\right][P]}}{2 [P]}{\Delta }_{\delta max}$$

where ${\Delta }_{\delta }$ is the change in chemical shift upon addition of ligand, [P] is the concentration of rhOSM, [L] is the concentration of ligand delivered, and ${\Delta }_{\delta max}$ is the maximum chemical change upon saturation of rhOSM with ligand. Curve fitting and error analysis was performed using Wolfram Mathematica to obtain a dissociation constant (*K*_*d*_).

### Fluorescence quenching

Fluorescence quenching measurements were made in triplicate solutions of 1 µM rhOSM (100 mM sodium chloride, 50 mM sodium phosphate, 0.1 mM DTT, pH 6.6) and titrated with approximately 5 µM increments using a 1.875 mM small molecule stock solution in DMSO. An additional 1 µM rhOSM solution was titrated with equivalent volumes of DMSO to account for changes in fluorescence due to dilution and solvent effects. A total of 16 titration points were collected not to exceed 5% (v/v) total DMSO. The SMI absorbance peaks were all less than 280 nm and did not absorb light at either the excitation or tryptophan emission wavelengths, so there was no need to correct for inner filter effects.

Fluorescence measurements were made on a Cary Eclipse spectrofluorometer equipped with a Xe arc lamp. Fluorescence intensities were measure by exciting 280 nm (4 nm slit width) and recording the emission from 345 to 355 nm with a photomultiplier tube voltage of 700 V. The fluorescence at each titration point was corrected by adding the decrease in fluorescence intensity caused by dilution with DMSO and normalized (***f***) to the fluorescence intensity in the absence of small molecule (***f***_***0***_). The average value of the three replicates for each point was plotted against concentration of small molecule delivered, and fit to a Stern–Volmer, modified Stern–Volmer, and rearranged modified Stern–Volmer equation, $\frac{f}{{f}_{0}}= \frac{{f}_{a}}{1-\frac{[X]}{Kd}}+(1-{f}_{a})$, originally described by Charlier et al.^67^, where $\frac{f}{{f}_{0}}$ is the fluorescence signal of OSM with SMI divided by the fluorescence signal of the blank, ${f}_{a}$ is the fraction of accessible fluorescence, [X] is the concentration of the SMI, and the dissociation constant (*K*_*d*_). *K*_*d*_ values were obtained from a least squares regression fitting routine and errors are reported as the standard error of the fit with a symmetrical 95% confidence interval. Raw data were analyzed using Microsoft Excel and all curve fitting analyses were performed in Prism 8 (v. 8.2.1, GraphPad Software).

## Supplementary Information

Supplementary Information.

## Data Availability

The datasets generated during and/or analysed during the current study are either available in the supplemental information or from the corresponding author on reasonable request.

## References

[1] Heinrich PC (2003). Principles of interleukin (IL)-6-type cytokine signalling and its regulation. Biochem. J..

[2] Bamber B, Reife RA, Haugen HS, Clegg CH (1998). Oncostatin M stimulates excessive extracellular matrix accumulation in a transgenic mouse model of connective tissue disease. J. Mol. Med..

[3] Larrea E (2009). Oncostatin M Enhances the Antiviral Effects of Type I Interferon and Activates Immunostimulatory Functions in Liver Epithelial Cells. J. Virol..

[4] Miyajima A (2000). Role of oncostatin M in hematopoiesis and liver development. Cytokine Growth Factor Rev..

[5] Elks C, Stephens J (2015). Oncostatin M modulation of lipid storage. Biology (Basel)..

[6] Sims NA, Quinn JMW (2014). Osteoimmunology: oncostatin M as a pleiotropic regulator of bone formation and resorption in health and disease. Bonekey Rep..

[7] West NR (2017). Oncostatin M drives intestinal inflammation and predicts response to tumor necrosis factor–neutralizing therapy in patients with inflammatory bowel disease. Nat. Med..

[8] Hui W, Rowan AD, Richards CD, Cawston TE (2003). Oncostatin M in combination with tumor necrosis factor α induces cartilage damage and matrix metalloproteinase expression in vitro and in vivo. Arthritis Rheum..

[9] Arunachalam PS (2020). Systems biological assessment of immunity to mild versus severe COVID-19 infection in humans. Science.

[10] Guo L (2013). Stat3-coordinated Lin-28-let-7-HMGA2 and miR-200-ZEB1 circuits initiate and maintain oncostatin M-driven epithelial-mesenchymal transition. Oncogene.

[11] Liang H (2012). Interleukin-6 and oncostatin M are elevated in liver disease in conjunction with candidate hepatocellular carcinoma biomarker GP73. Cancer Biomark..

[12] Royuela M (2004). Immunohistochemical analysis of the IL-6 family of cytokines and their receptors in benign, hyperplasic, and malignant human prostate. J. Pathol..

[13] Holzer RG (2004). Oncostatin M stimulates the detachment of a reservoir of invasive mammary carcinoma cells: Role of cyclooxygenase-2. Clin. Exp. Metastasis.

[14] Jorcyk CL, Holzer RG, Ryan RE (2006). Oncostatin M induces cell detachment and enhances the metastatic capacity of T-47D human breast carcinoma cells. Cytokine.

[15] Holzer RG (2003). Development and characterization of a progressive series of mammary adenocarcinoma cell lines derived from the C3(1)/SV40 Large T-antigen transgenic mouse model. Breast Cancer Res. Treat..

[16] Tawara K (2019). Co-expression of VEGF and IL-6 family cytokines is associated with decreased survival in HER2 negative breast cancer patients: subtype-specific IL-6 family cytokine-mediated VEGF secretion. Transl. Oncol..

[17] Vollmer S (2009). Hypoxia-inducible factor 1α is up-regulated by oncostatin M and participates in oncostatin M signaling. Hepatology.

[18] Bolin C (2012). Oncostatin M promotes mammary tumor metastasis to bone and osteolytic bone degradation. Genes Cancer.

[19] Tawara K (2018). OSM potentiates preintravasation events, increases CTC counts, and promotes breast cancer metastasis to the lung. Breast Cancer Res..

[20] Tawara K (2019). HIGH expression of OSM and IL-6 are associated with decreased breast cancer survival: synergistic induction of IL-6 secretion by OSM and IL-1β. Oncotarget.

[21] Liu J (1998). Oncostatin M-specific receptor expression and function in regulating cell proliferation of normal and malignant mammary epithelial cells. Cytokine.

[22] Hermanns HM (2015). Oncostatin M and interleukin-31: cytokines, receptors, signal transduction and physiology. Cytokine Growth Factor Rev..

[23] Rhodes A (2000). The generation and characterization of antagonist RNA aptamers to human oncostatin M. J. Biol. Chem..

[24] Reid J (2018). In vivo affinity and target engagement in skin and blood in a first-time-in-human study of an anti-oncostatin M monoclonal antibody. Br. J. Clin. Pharmacol..

[25] Pöling J (2014). Therapeutic targeting of the oncostatin M receptor-β prevents inflammatory heart failure. Basic Res. Cardiol..

[26] Kucia-Tran JA (2018). Anti-oncostatin M antibody inhibits the pro-malignant effects of oncostatin M receptor overexpression in squamous cell carcinoma. J. Pathol..

[27] Mócsai A, Kovács L, Gergely P (2014). What is the future of targeted therapy in rheumatology: Biologics or small molecules?. BMC Med..

[28] Deller MC (2000). Crystal structure and functional dissection of the cytostatic cytokine oncostatin M. Structure.

[29] Nair BC (1992). Identification of a major growth factor for AIDS-Kaposi’s sarcoma cells as oncostatin M. Science.

[30] Linsley PS, Kallestad J, Ochs V, Neubauer M (1990). Cleavage of a hydrophilic C-terminal domain increases growth-inhibitory activity of oncostatin M. Mol. Cell. Biol..

[31] Adrian-Segarra JM (2018). The AB loop and D-helix in binding site III of human Oncostatin M (OSM) are required for OSM receptor activation. J. Biol. Chem..

[32] Shuker SB, Hajduk PJ, Meadows RP, Fesik SW (1996). Discovering high-affinity ligands for proteins: SAR by NMR. Science.

[33] Ashkenazi A, Fairbrother WJ, Leverson JD, Souers AJ (2017). From basic apoptosis discoveries to advanced selective BCL-2 family inhibitors. Nat. Rev. Drug Discov..

[34] Bessette PH, Åslund F, Beckwith J, Georgiou G (1999). Efficient folding of proteins with multiple disulfide bonds in the Escherichia coli cytoplasm. Proc. Natl. Acad. Sci. U.S.A..

[35] Siligardi G, Hussain R (2015). CD spectroscopy: an essential tool for quality control of protein folding. Methods Mol. Biol..

[36] Cole DR, Stein WH, Moore S (1958). On the cysteine content of human hemoglobin. J. Biol. Chem..

[37] Nguyen MT (2019). Prokaryotic soluble overexpression and purification of oncostatin M using a fusion approach and genetically engineered E coli strains. Sci. Rep..

[38] Huynh K, Partch CL (2015). Analysis of protein stability and ligand interactions by thermal shift assay. Curr. Protoc. Protein Sci..

[39] Golovanov AP, Hautbergue GM, Wilson SA, Lian L-Y (2004). A simple method for improving protein solubility and long-term stability. J. Am. Chem. Soc..

[40] Williamson MP (2013). Using chemical shift perturbation to characterise ligand binding. Prog. Nucl. Magn. Reson. Spectrosc..

[41] Miller Z (2015). Proteasome inhibitors with pyrazole scaffolds from structure-based virtual screening. J. Med. Chem..

[42] Sterling T, Irwin JJ (2015). ZINC 15—ligand discovery for everyone. J. Chem. Inf. Model..

[43] Jorcyk, C. & Xu, D. Oncostatin M (OSM) antagonists for preventing cancer metastasis and IL-6 related disorders. US Patent 10286070 (May 14, 2019).

[44] Jorcyk, C. L., Warner, D. L., King, M. D. & Warner, L. R. Pharmaceutical compositions comprising oncostatin m (osm) antagonist derivatives and methods of use. US Patent Application 20200323960 (October 15, 2020).

[45] Hoffman R (1996). Resonance assignments for Oncostatin M, a 24-kDa α-helical protein. J. Biomol. NMR.

[46] Scott DE, Bayly AR, Abell C, Skidmore J (2016). Small molecules, big targets: Drug discovery faces the protein-protein interaction challenge. Nat. Rev. Drug Discov..

[47] Barile E, Pellecchia M (2014). NMR-based approaches for the identification and optimization of inhibitors of protein-protein interactions. Chem. Rev..

[48] Juárez-Salcedo LM, Desai V, Dalia S (2019). Venetoclax: Evidence to date and clinical potential. Drugs Context.

[49] Emerson SD (2003). NMR characterization of interleukin-2 in complexes with the IL-2Ralpha receptor component, and with low molecular weight compounds that inhibit the IL-2/IL-Ralpha interaction. Protein Sci..

[50] Sporeno E (1994). Production and structural characterization of amino terminally histidine tagged human oncostatin M in E. Coli. Cytokine.

[51] Kallestad JC, Shoyab M, Linsley PS (1991). Disulfide bond assignment and identification of regions required for functional activity of oncostatin M. J. Biol. Chem..

[52] Drechsler J, Grötzinger J, Hermanns HM (2012). Characterization of the rat Oncostatin M receptor complex which resembles the human, but differs from the murine cytokine receptor. PLoS ONE.

[53] Stead P (2000). Discovery of novel ansamycins possessing potent inhibitory activity in a cell-based oncostatin M signalling assay. J. Antibiot. (Tokyo).

[54] Life, P. F. Oncostatin M antagonists as inflammatory mediator antagonists. *PCT Int. Appl.* WO 99/48523 (September 30, 1999).

[55] Harner MJ, Frank AO, Fesik SW (2013). Fragment-based drug discovery using NMR spectroscopy. J. Biomol. NMR.

[56] Shortridge MD, Hage DS, Harbison GS, Powers R (2008). Estimating protein-ligand binding affinity using high-throughput screening by NMR. J. Comb. Chem..

[57] Mayer M, Meyer B (1999). Characterization of ligand binding by saturation transfer difference NMR spectroscopy. Angew. Chemie Int. Ed..

[58] Whitmore L, Wallace BA (2004). DICHROWEB, an online server for protein secondary structure analyses from circular dichroism spectroscopic data. Nucleic Acids Res..

[59] Lees JG, Miles AJ, Wien F, Wallace BA (2006). A reference database for circular dichroism spectroscopy covering fold and secondary structure space. Bioinformatics.

[60] Waterhouse A (2018). SWISS-MODEL: homology modelling of protein structures and complexes. Nucleic Acids Res..

[61] Drew ED, Janes RW (2020). PDBMD2CD: providing predicted protein circular dichroism spectra from multiple molecular dynamics-generated protein structures. Nucleic Acids Res..

[62] Wilkins MR (1997). Detailed peptide characterization using PEPTIDEMASS—a world-wide-web-accessible tool. Electrophoresis.

[63] Trott O, Olson AJ (2010). AutoDock Vina: Improving the speed and accuracy of docking with a new scoring function, efficient optimization, and multithreading. J. Comput. Chem..

[64] Piotto M, Saudek V, Sklenář V (1992). Gradient-tailored excitation for single-quantum NMR spectroscopy of aqueous solutions. J. Biomol. NMR.

[65] Delaglio F (1995). NMRPipe: a multidimensional spectral processing system based on UNIX pipes. J. Biomol. NMR.

[66] Helmus JJ, Jaroniec CP (2013). Nmrglue: an open source python package for the analysis of multidimensional NMR data. J. Biomol. NMR.

[67] Charlier HA, Plapp BV (2000). Kinetic cooperativity of human liver alcohol dehydrogenase γ2. J. Biol. Chem..

