# Supplementary material for: Bioactive recombinant human oncostatin M for NMR-based screening in drug discovery
Source: Sci Rep. 2021 Aug 10;11:16174. doi: 10.1038/s41598-021-95424-6 (PMC8355150; doi:10.1038/s41598-021-95424-6)
Supplement: Supplementary file 1 — Supplementary Information. [file 41598_2021_95424_MOESM1_ESM.pdf]

Supporting Information for

Bioactive Recombinant Human Oncostatin M for NMR-based Screening in Drug Discovery

Olga A. Mass[1#], Joseph Tuccinardi[2#], Luke Woodbury[1], Cody L. Wolf[3], Bri Grantham[1], Kelsey Holdaway[2], Xinzhu Pu[1,3], Matthew D. King[2,3], Don L. Warner[2,3], Cheryl L. Jorcyk[3,4], Lisa R. Warner[1,2,3*]

[1]Biomoleculer Research Center, Boise State University, ID 83725
[2]Department of Chemistry and Biochemistry, Boise State University, ID 83725
[3]Biomolecular Sciences Graduate Program, Boise State University, ID, 83725
[4]Department of Biological Sciences, Boise State University, ID 83725
[#]These authors contributed equally

*Corresponding Author:
Lisa R. Warner
Department of Chemistry and Biochemistry
Boise State University
1910 University Dr.
Boise, ID 83725-1520
208-426-2260
lisawarner@boisestate.edu

Table of Contents

Table of Contents 1
Table of Figures 2
Table of Tables 2
Table of Schemes 2
Expression optimization of 6-His-OSM 3
Solubility of 6-His-OSM 4
MBP-OSM expression and solubility 5
Addition of algal lysate decreases time to induction 5
Purification of ^{15}N MBP-OSM 6
Replicate expression and purification of MBP-OSM 6
Mass spectrometry analysis of rhOSM 13
Protein long-term stability assay 19
Chemistry 20
Fluorescence Quenching 30
References 33

Table of Figures

Figure S1. 6-His-OSM Test Expressions. 3
Figure S2. Effects of glucose (C6) on 6-His-OSM solubility in BL21(DE3) and SHuffle *E. coli*. 4
Figure S3. Expression and solubility of MBP-OSM. 5
Figure S4. Addition of 0.5 g/L algal lysate decreases time to induction. 5
Figure S5. Expression and purification SDS-PAGE gels of ^{15}N MBP-OSM. 6
Figure S6. Purification of rhOSM using amylose affinity chromatography. 7
Figure S7. Purification of rhOSM using ion exchange chromatography. 8
Figure S8. SDS-PAGE gels of expression and purification rhOSM. 9
Figure S9. Purification of rhOSM with size exclusion chromatography. 10
Figure S10. SDS-PAGE gels of size exclusion chromatography purification of rhOSM. 11
Figure S11. Western blot of STAT3 induction by OSM. 12
Figure S12. Sequence coverage of purified rhOSM from MBP-OSM expression and purification. 13
Figure S13. MALDI-TOF spectrum of rhOSM tryptic digest. 13
Figure S14. MALDI-TOF spectrum of alkylated rhOSM tryptic digest. 14
Figure S15. Effect of additives on long-term rhOSM stability. 19
Figure S16. ^{1}H NMR (600 MHz, $CDCl_3$) aldehyde 2 22
Figure S17. ^{1}H NMR (600 MHz, $CDCl_3$) aldehyde 2 (inset) 23
Figure S18. ^{13}C NMR (151 MHz, $CDCl_3$) aldehyde 2 24
Figure S19. ^{1}H NMR (600 MHz, DMSO-d6) OSM-SMI-10B 25
Figure S20. ^{1}H NMR (600 MHz, DMSO-d6) OSM-SMI-10B (inset). 26
Figure S21. ^{13}C NMR (600 MHz, DMSO-d6) OSM-SMI-10B 27
Figure S22. HRMS and MS/MS of $[M+H]^+$ of OSM-SMI-10B 28
Figure S23. HPLC Chromatogram of OSM-SMI-10B 29
Figure S24. Quenching of rhOSM tryptophan 187 fluorescence by OSM-SMI-10B. 31
Figure S25. Comparison of fluorescence quenching and NMR titration data for OSM-SMI-10B11 and OSM-SMI-27A6 with OSM-SMI-10B. 32

Table of Tables

Table S1. Yields from rhOSM expression and purification (n=3). 6
Table S2. MALDI-TOF peak list of rhOSM tryptic digest. 15
Table S3. MALDI-TOF peak list of alkylated rhOSM tryptic digest. 17

Table of Schemes

Scheme S1. Synthesis of OSM-SMI-10B 20

Expression optimization of 6-His-OSM

Our initial attempts to express OSM in BL21(DE3) host were characterized by inconsistent expression despite normal cell growth before and after induction. Specifically, freshly transformed cells were growing well before and after induction with IPTG in LB or defined minimal growth media (M9) reaching high optical density, however, the expression of OSM was unpredictable with yield varying from zero to modest. Well known methods of induction regulation like change of temperature and concentration of IPTG (0.1mM, 0.5 mM, 0.7 mM and 1.0 mM at 18 °C) did not influence the inconsistent expression. Methods reducing basal expression of a toxic protein (pLysS host, 1-2% glucose (labeled as C6 in the figures) in growth medium) proved to be ineffective as well. Based on these observations we excluded OSM toxicity as the source of unpredictable expression and reasoned that misfolding of OSM was causing proteolytic degradation by host proteases. Such proteolytic digestion is especially commonly observed upon overexpression of heterogeneous proteins with multiple disulfide bonds[1]. The M9 minimal media has been suggested to reduce levels of *E. coli* endogenous proteases,[2] but alone did not improve the OSM expression in our case. However, we noticed that supplementing minimal growth media with high concentration of glucose (150 mM), guaranteed OSM expression in BL21 with moderate yield (**Fig. S1A & S1C**). A possible explanation is that here glucose acted as a chemical chaperone stabilizing protein in a folded state. By analogy, osmolytes such as sorbitol and ethylene glycol have been previously utilized in the expression of recombinant proteins to increase an amount of soluble protein in *E. coli*[3]. As an alternative method we tested autoinduction which afforded OSM in the yield comparable to the expression in M9/glucose (**Fig. S1B**). Nevertheless, addition of glucose and autoinduction still did not appear as feasible strategies for isotopically labeled protein production. It is noted that while we routine use a low level of algal lysate in large scale growth to decrease the time to induction (**Fig. S4**), in the initial expression optimizations shown in Figs S1 & S2, algal lysate (Isogro) was not used.

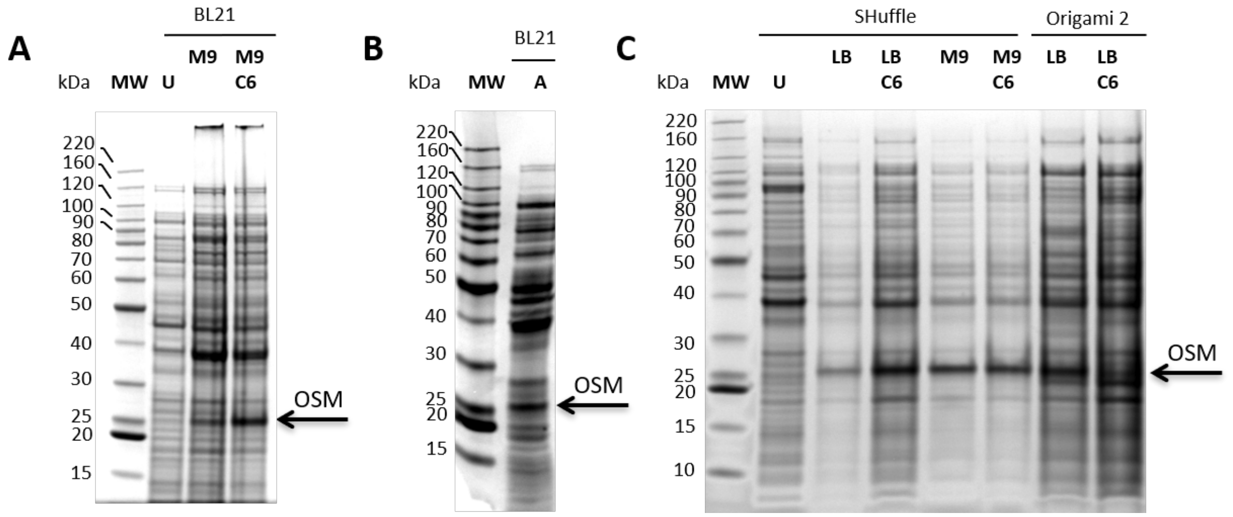

Figure S1. 6-His-OSM Test Expressions.
(**A**) Effect of glucose (C6) on 6His-OSM expression in BL21 (DE3). Note that OSM sample induced in M9 is a representative example when a low yield of OSM expression was observed. (**B**) 6His-OSM expression in BL21 in autoinduction medium. (**C**) 6His-OSM expression in SHuffle and Origami 2. All samples except autoinduction were induced with 1.0 mM IPTG followed by expression overnight at 18 °C. *Lane labels*: MW – molecular weight marker; U – OSM pre-induced; LB – OSM post-induced in LB, M9 – OSM post-induced in M9; M9/C6 – OSM post-induced in M9/150 mM glucose, A – OSM post-induced in autoinduction medium; LB – OSM post-induced in LB; LB/C6 – OSM post-induced in LB/150 mM glucose.

Solubility of 6-His-OSM

To estimate the amount of soluble OSM expressed under different conditions, cell pellets were subjected to three freeze-thaw cycles after which a pellet and a supernatant were separated and analyzed by SDS-PAGE. The relative amounts of pellet and soluble fraction were calculated by densitometric analysis using ImageJ software. The ratio of OSM protein in the pellet:supernatant was estimated to be 2:1 in BL21 M9/C6 (**Fig. S2A**), and 1:1 in SHuffle LB, SHuffle LB/C6 and SHuffle M9 (**Fig. S2B**).

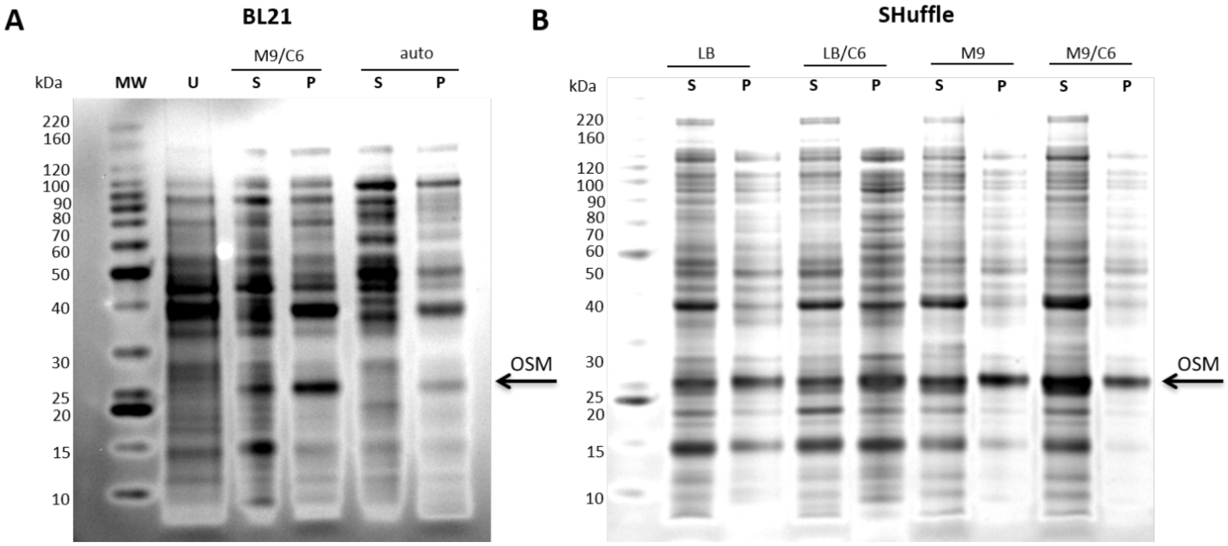

Figure S2. Effects of glucose (C6) on 6-His-OSM solubility in BL21(DE3) and SHuffle *E. coli*.
(**A**) Soluble and insoluble fractions of OSM expressed in BL21(DE3) in M9 with 150 mM glucose and autoinduction media. (**B**) Soluble and insoluble fractions of OSM expressed in Shuffle in LB and M9 (with and without supplementation with 150 mM glucose (C6)). All samples except autoinduction were induced with 1.0 mM IPTG followed by overnight expression at 18 °C. *Lane labels*: MW – molecular weight marker; U – pre-induced OSM; S – soluble fraction; P – pellet or insoluble fraction; M9 – OSM expressed in M9; M9/C6 – OSM expressed in M9 with 150 mM glucose; auto – OSM expressed via autoinduction; LB – OSM expressed in LB; LB/C6 – OSM expressed in LB with 150 mM glucose.

MBP-OSM expression and solubility

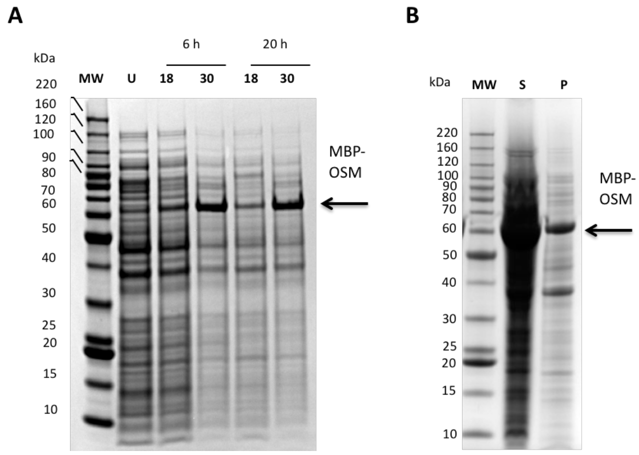

Figure S3. Expression and solubility of MBP-OSM.
(**A**) MBP-OSM induction in SHuffle grown in LB at 18 ºC and at 30 °C overnight. (**B**) MBP-OSM in pellet and soluble fraction after lysis by sonication. *Lane labels*: MW – molecular weight; U – uninduced; I – induced; S – soluble fraction of OSM; P – pellet.

Addition of algal lysate decreases time to induction

Minimal defined medium (M9) supplemented with algal lysates at low levels (0.5 g/L) increases the growth rate of *E. coli* in minimal media (decreases the time to induction). This adds a nominal cost to the overall culture ($30 – $50/L).

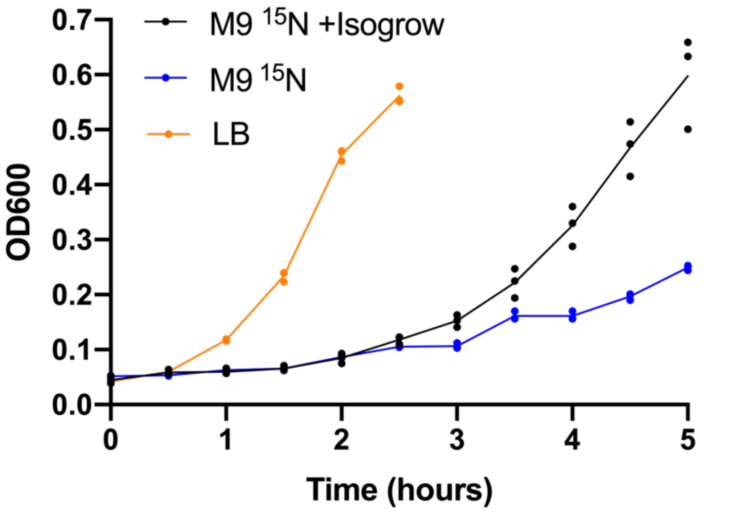

Figure S4. Addition of 0.5 g/L algal lysate decreases time to induction.
Growth curve for SHuffle cells transformed with the MBP-OSM plasmid grown in LB (orange), ^{15}N M9 medium (blue) or ^{15}N M9 medium supplemented with 0.5 g/L ^{15}N algal lysate (black). All time points were collected on n=3 cultures. Replicates are plotted.

Purification of ^{15}N MBP-OSM

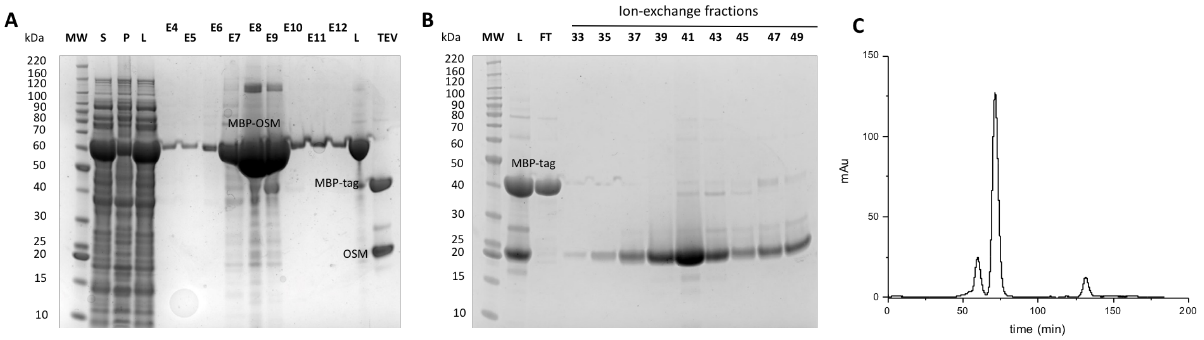

Figure S5. Expression and purification SDS-PAGE gels of ^{15}N MBP-OSM.
(A) SDS-PAGE showing purification of ^{15}N MBP-OSM by amylose affinity chromatography and cleavage with TEV. (**B**) SDS-PAGE showing purification of TEV cleaved ^{15}N MBP-OSM. (**C**) Size-exclusion chromatogram of ^{15}N OSM. *Lane labels*: MW – molecular weight marker; S – soluble fraction; P – pellet or insoluble fraction, L – load for ion-exchange column; FT – column flow through; E – elution fractions

Replicate expression and purification of MBP-OSM

We performed expression and purification of rhOSM from rich and minimal defined, isotope enriched (^{15}N, and ^{13}C ,^{15}N) media in triplicate. To balance time and cost, the replicates were performed in 100 mL cultures. The yields were lower (2–3 mg/L of culture) than what we routinely obtain from 1 L cultures (8.6 ± 1.9 mg/L of culture over 7 preps; both LB and ^{15}N). The loss occurred at the amylose affinity column step, which is clear from the SDS-PAGE gels (**Fig. S7**). We suspect that this was due to a new batch of amylose resin and smaller bead volumes, which changed the dynamic binding capacity of the amylose resin, leading to breakthrough of the MBP-OSM. What is clear however, is that even when breaking in a new resin, there is low variation in protein yields between replicates.

Table S1. Yields from rhOSM expression and purification (n=3).

Lysogeny Broth (mg/L culture)	M9 ^{15}N + 0.5% ^{15}N Isogro (mg/L culture)	M9 ^{13}C, ^{15}N + 0.5% ^{13}C, ^{15}N Isogro (mg/L culture)
2.6 ± 0.8	2.7 ± 0.5	2.2 ± 0.7

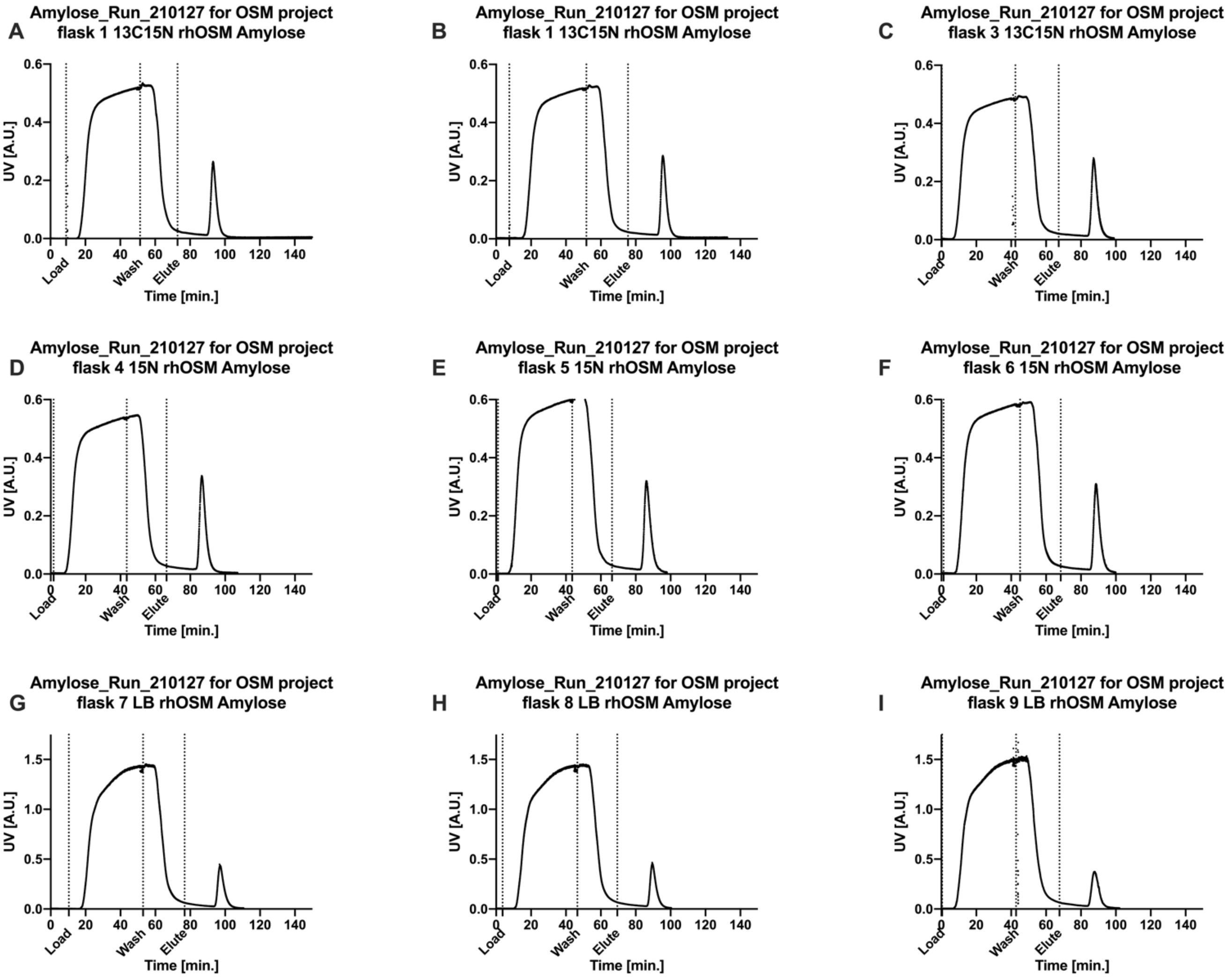

Figure S6. Purification of rhOSM using amylose affinity chromatography.
Chromatograms from amylose affinity chromatography experiments showing the initial purification of ^{13}C,^{15}N enriched rhOSM (**A-C**), ^{15}N enriched rhOSM (**D-F**), and unenriched rhOSM (**G-I**). Input material was loaded onto amylose resin and eluted using a linear gradient of 0 – 10 mM maltose. Vertical lines indicate load (where input material was loaded onto the column), wash (where wash buffer was run over the column after input material was loaded), and gradient (where the elution gradient program was initiated). Purified rhOSM peaks elute between 80 and 120 minutes.

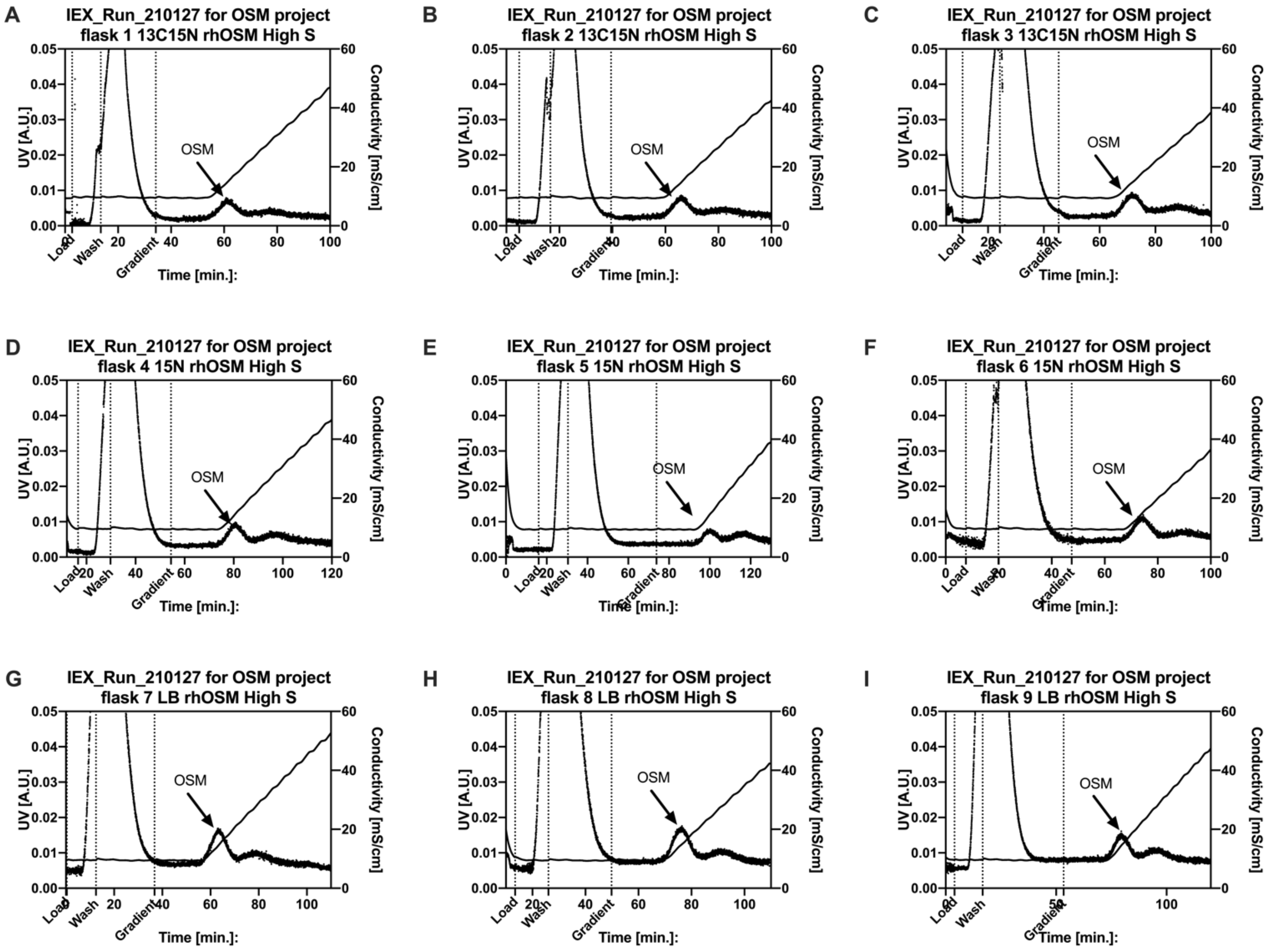

Figure S7. Purification of rhOSM using ion exchange chromatography.
Chromatograms from ion exchange chromatography (IEX) experiments showing the subsequent purification of ^{13}C,^{15}N enriched rhOSM (**A-C**), ^{15}N enriched rhOSM (**D-F**), and unenriched rhOSM (**G-I**). Fractions from amylose affinity chromatography experiments were pooled, incubated overnight with TEV protease, and dialyzed against PBS containing 10mM NaCl. The material was then loaded onto Macroprep high S resin and eluted with an increased gradient of salt. Vertical lines indicate load (where input material was loaded onto the column), wash (where low salt buffer was run over the column after input material was loaded), and gradient (where the elution gradient program was initiated). Peaks containing rhOSM are shown.

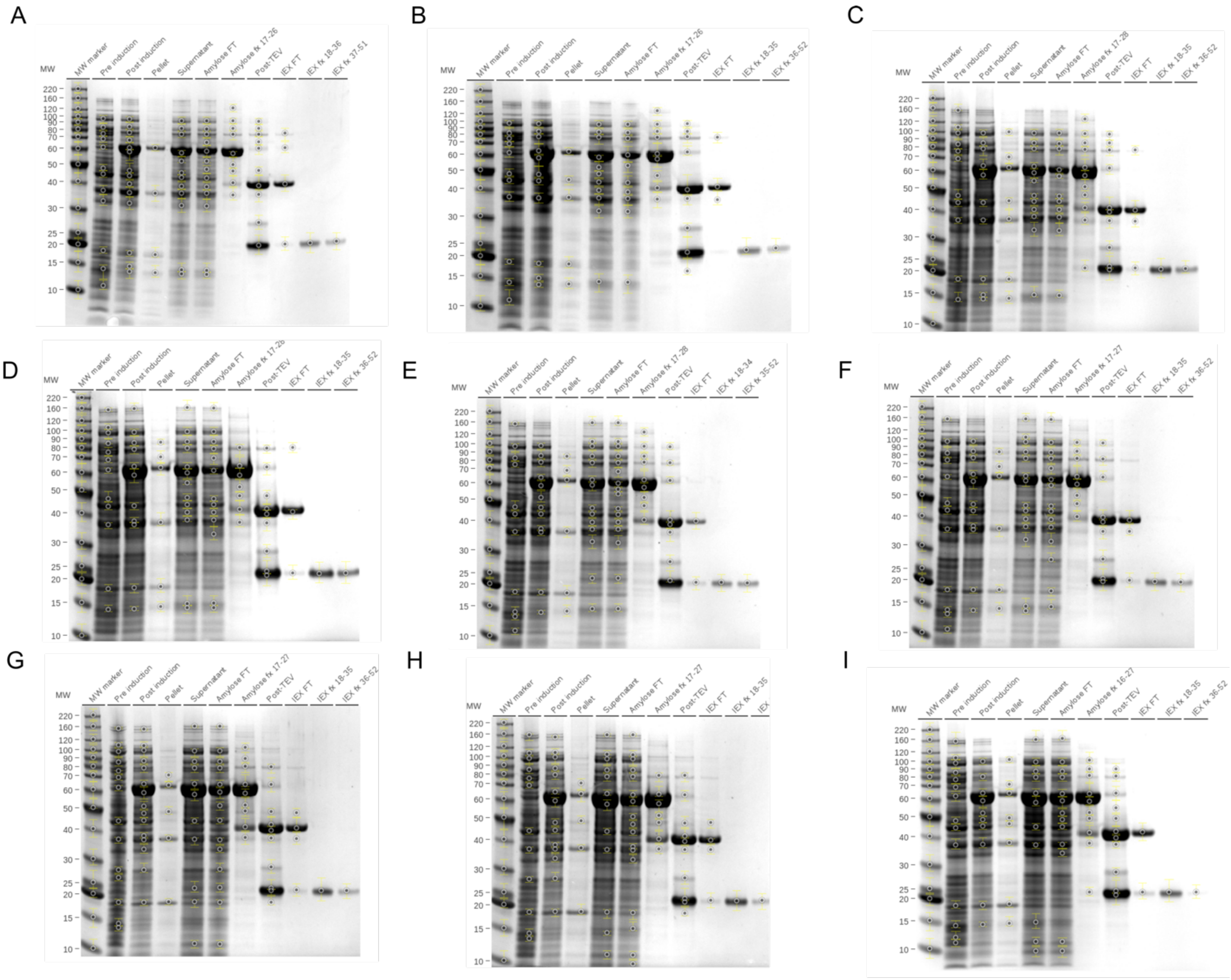

Figure S8. SDS-PAGE gels of expression and purification rhOSM.
SDS-PAGE of expression and purification steps for $^{13}C^{15}N$ enriched rhOSM (**A-C**), ^{15}N enriched rhOSM **(D-F)**, and unenriched rhOSM **(G-I)**. *Lane labels*: MW marker – molecular weight marker; Pre induction – pMBP-OSM Shuffle before induction with IPTG; Post induction – pMBP-OSM shuffle after 18h induction with 1mM IPTG; Pellet – insoluble fraction; Supernatant – soluble fraction; Amylose FT – amylose column flow through; Amylose fx – amylose fractions pooled for IEX purification experiments; Post-TEV – after overnight TEV cleavage; IEX FT – ion exchange column flow through; IEX fx – ion exchange fractions pooled for size exchange chromatography experiments.

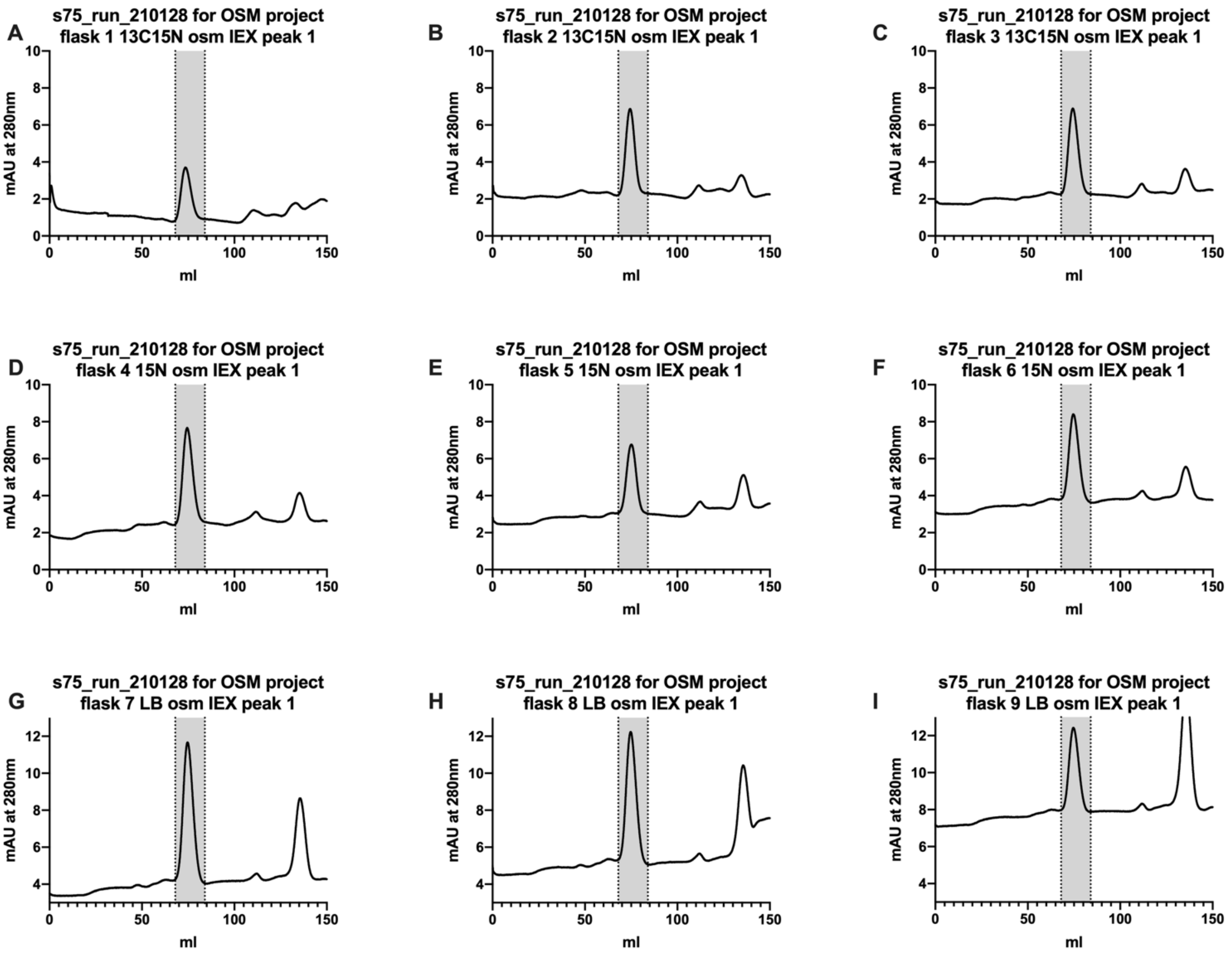

Figure S9. Purification of rhOSM with size exclusion chromatography.
Chromatograms from size exclusion chromatography (SEC) experiments showing the subsequent purification of ^{13}C,^{15}N enriched rhOSM **(A-C)**, ^{15}N enriched rhOSM **(D-F)**, and unenriched rhOSM **(G-I)**. Fractions from ion exchange chromatography experiments were pooled, concentrated, then loaded onto an S75 column and eluted with 2 CVs of NMR buffer. Vertical lines and greyed area indicate rhOSM fractions that were run on SDS-PAGE gels.

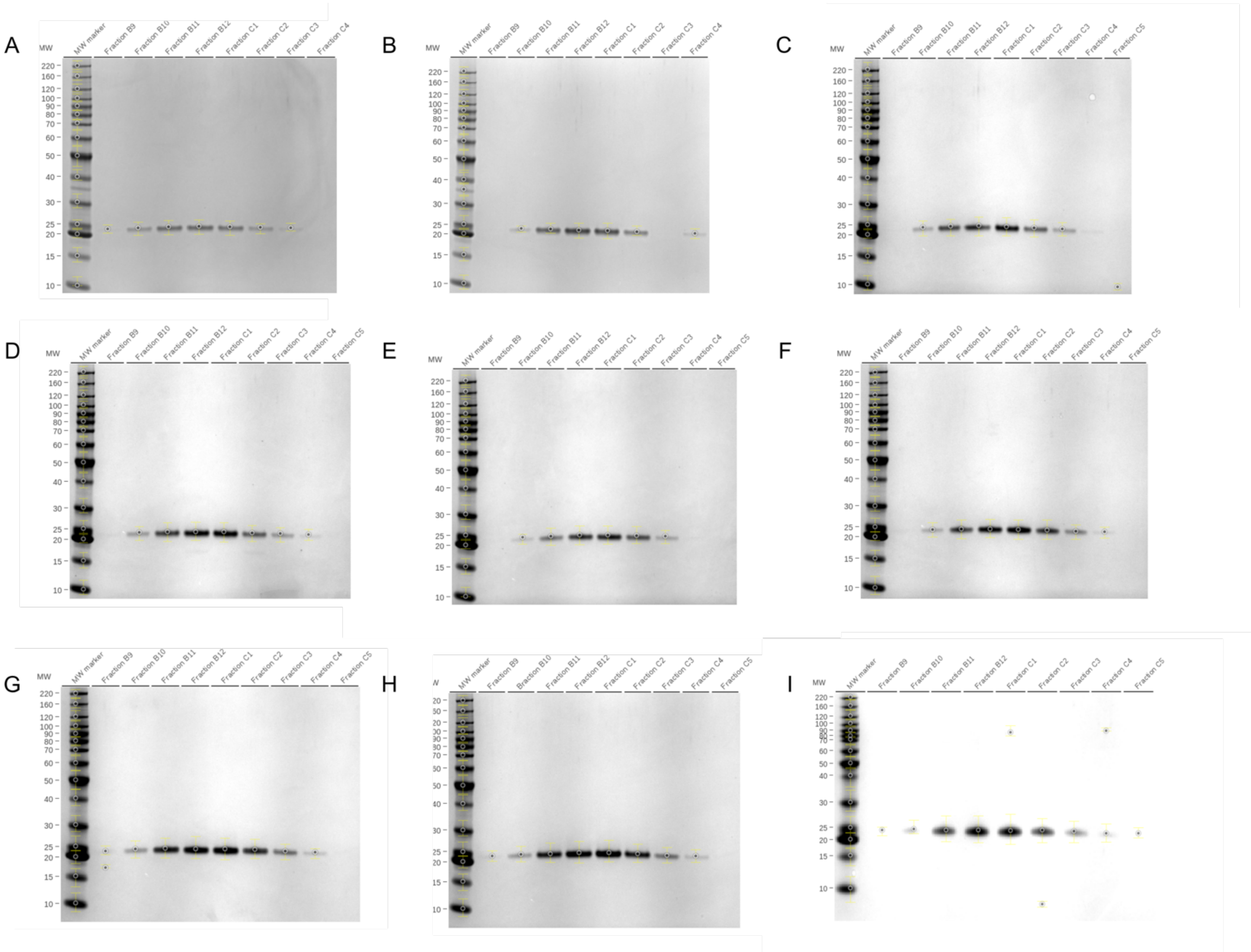

Figure S10. SDS-PAGE gels of size exclusion chromatography purification of rhOSM.
SDS-PAGE of fractions collected from the SEC peaks of $^{13}C^{15}N$ enriched rhOSM (**A-C**), ^{15}N enriched rhOSM (**D-E**), and unenriched rhOSM (**G-I**). *Lane labels*: MW marker – molecular weight marker; Fraction # – fraction from peak on SEC chromatogram.

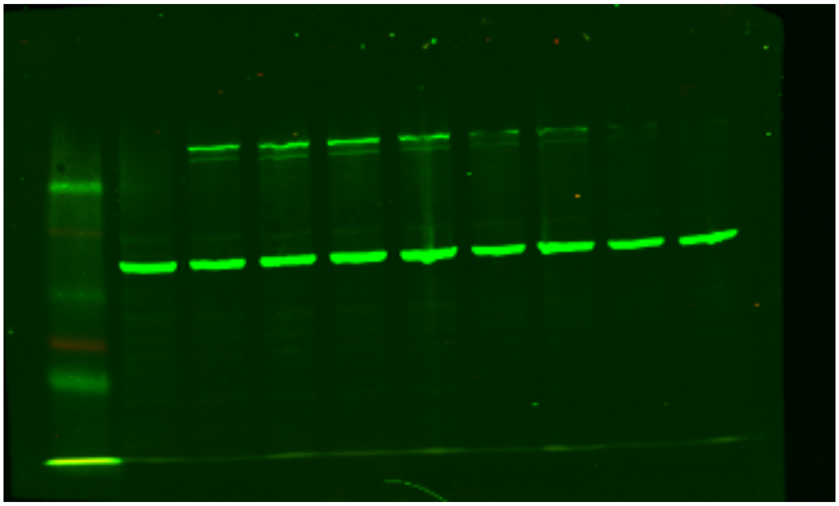

Figure S11. Western blot of STAT3 induction by OSM.
T47D cells were untreated (NT), treated with commercial OSM (chOSM) or treated with increasing concentrations of recombinant OSM (rhOSM) for 30 minutes and levels of phosphorylated STAT3 (PSTAT3;Y705, upper band) were evaluated via immunoblot assay, normalized to beta-actin (lower band). *Lanes: 1, Molecular weight marker; 2, no treatment; 3, chOSM 25 ng/mL; 4, rhOSM 100 ng/mL; 4, rhOSM 100 ng/mL; 5, rhOSM 50 ng/mL; 6, rhOSM 25 ng/mL; 7, rhOSM 10 ng/mL; 8, rhOSM 5 ng/mL; 9, rhOSM 2.5 ng/mL; 10, rhOSM 1.25 ng/mL*

Mass spectrometry analysis of rhOSM

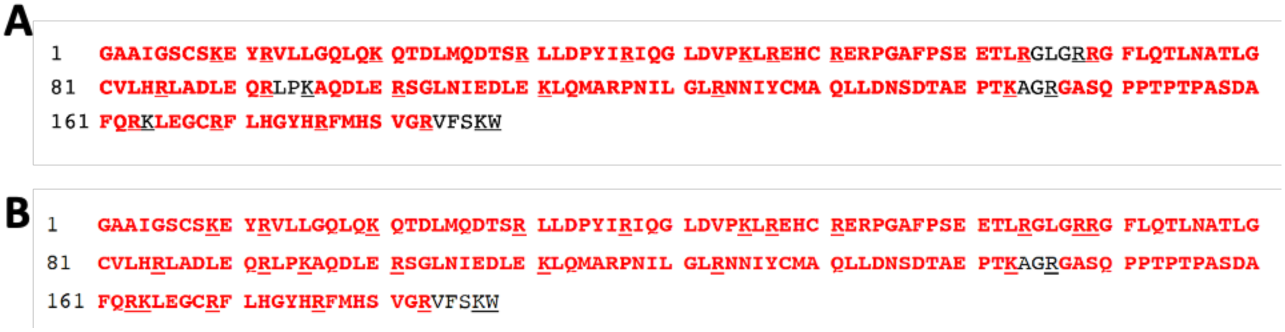

Figure S12. Sequence coverage of purified rhOSM from MBP-OSM expression and purification.
(A) Peptides identified from the MADLI-TOF mass spectrum covered 96% of the sequence of OSM in the absence of IAA and (B) 91% in the presence of IAA.

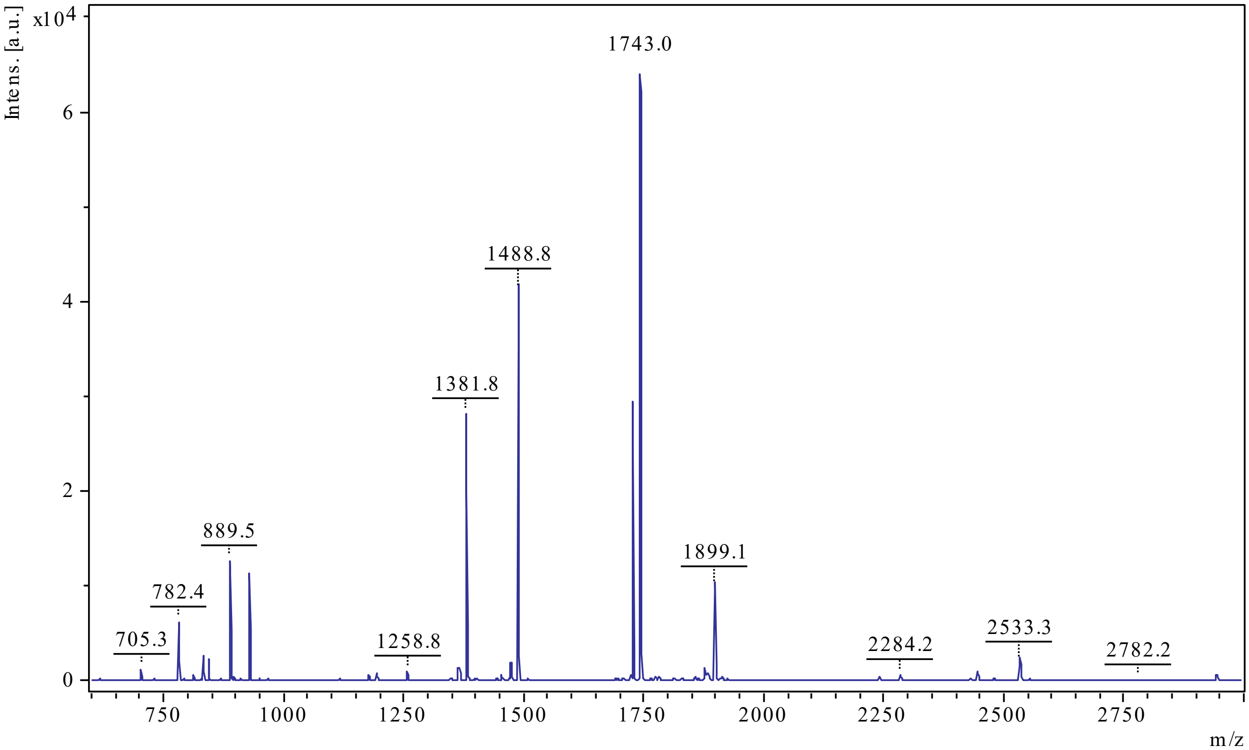

Figure S13. MALDI-TOF spectrum of rhOSM tryptic digest.
rhOSM did not undergo reduction and alkylation prior to tryptic digestion.

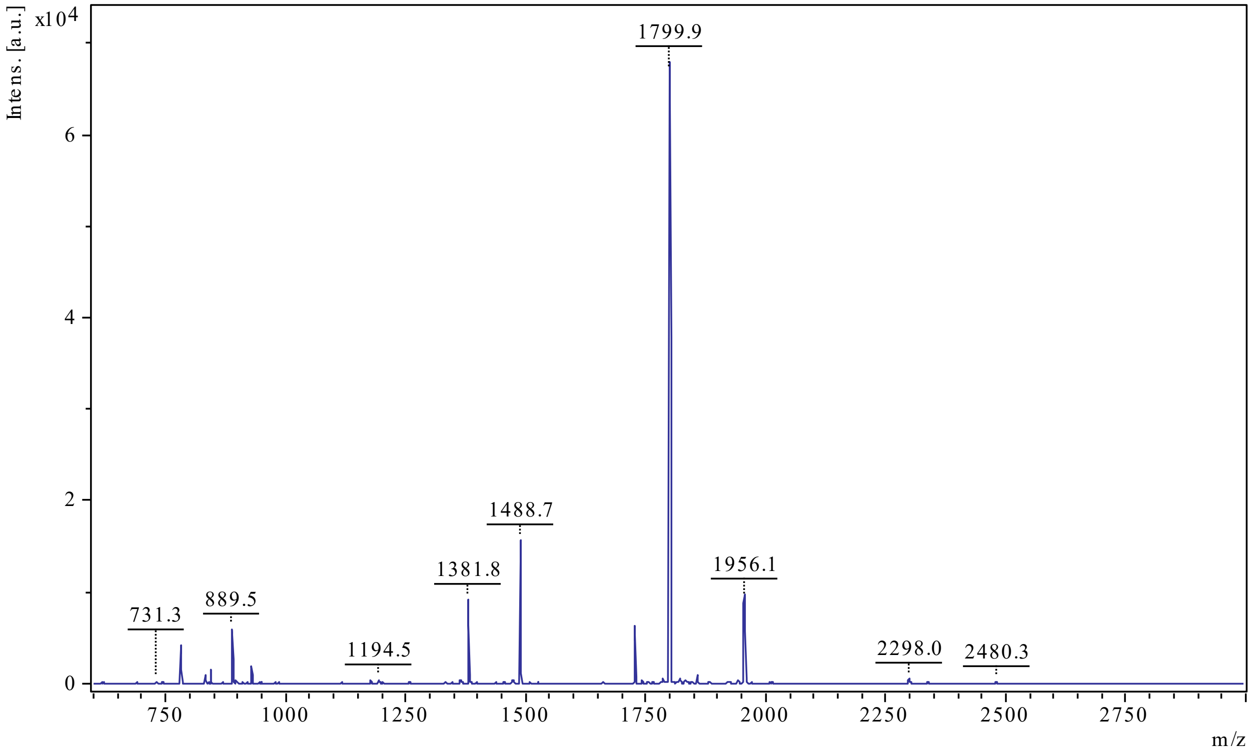

Figure S14. MALDI-TOF spectrum of alkylated rhOSM tryptic digest.
rhOSM was reduced with dithiothreitol and alkylated with iodoacetamide prior to tryptic digestion.

Table S2. MALDI-TOF peak list of rhOSM tryptic digest.
rhOSM did not undergo reduction and alkylation prior to tryptic digestion.

m/z	Peak Intensity	Signal to Noise Ratio	Peak Resolution	Peak Area
618.163	319.1	11.4	4323.3	71.7
705.268	1181.1	42.4	4653.0	295.9
731.268	265.6	9.5	3817.0	86.9
782.428	6078.7	218.8	5045.8	1589.1
793.328	154.7	5.6	3881.0	59.6
813.368	678.9	24.5	4581.6	211.5
829.378	188.4	6.8	4778.9	55.7
833.373	2625.3	94.4	5138.6	734.0
844.416	2260.6	81.3	5065.3	658.1
869.467	146.8	5.2	4379.0	54.9
889.497	12515.2	444.9	5323.7	3703.4
898.541	402.8	14.3	5040.4	131.3
911.470	166.4	5.9	5543.1	49.0
929.475	11269.1	397.7	5529.9	3421.2
951.451	144.1	5.1	4510.9	62.0
969.512	173.9	6.2	5237.2	61.0
1117.581	193.0	6.6	5211.0	87.1
1177.529	612.6	20.5	5545.8	272.1
1194.553	710.4	23.7	5545.5	325.2
1258.833	846.1	27.6	1697.6	1373.9
1348.038	270.5	8.5	2210.7	380.2
1365.593	1075.1	33.8	1584.2	2525.2
1368.063	1003.2	31.4	5404.9	592.1
1381.833	27962.6	874.5	6004.3	15129.0
1397.813	213.4	6.6	5315.1	139.9
1444.759	197.7	6.1	1883.9	387.4
1454.954	441.7	13.5	2807.6	561.2
1470.720	294.0	8.9	4991.3	230.6
1473.177	403.8	12.3	16261.4	99.8
1474.996	1601.3	48.7	3880.9	1542.8
1488.763	40808.6	1237.6	6140.6	24819.9
1722.908	652.7	20.0	6340.4	510.4
1727.849	28728.5	882.0	6582.4	22015.8
1742.956	65298.3	2016.2	6225.3	53672.1
1764.901	213.9	6.6	5323.9	215.0
1775.093	428.6	14.3	2269.8	1066.3

1782.292	133.2	6.4	546.1	1377.4
1812.977	283.7	8.9	5500.6	291.9
1830.149	162.5	6.0	1320.1	690.2
1857.201	150.1	6.6	1008.0	866.5
1876.941	1295.5	42.0	6394.0	1188.3
1879.005	436.9	14.2	17191.7	159.9
1879.990	228.4	7.7	5005.6	310.4
1884.188	430.4	18.6	1612.7	1713.6
1887.179	173.1	6.3	4262.2	295.9
1899.063	9666.1	318.7	6638.7	8650.3
1908.969	286.1	9.5	5927.4	298.7
1914.500	224.8	9.0	1180.3	1142.0
1925.049	155.2	5.2	6229.7	150.3
2241.004	357.1	16.7	6397.5	410.1
2284.151	427.3	20.9	5899.7	557.3
2431.315	88.1	5.0	3005.9	238.7
2445.299	717.7	41.0	6502.5	913.8
2480.363	189.0	11.1	5218.3	311.1
2533.333	1616.2	99.6	7435.9	1878.7
2553.314	111.5	7.0	5408.4	188.5
2782.222	69.3	5.5	5575.6	130.8
2943.356	413.7	36.9	8012.6	569.2
3482.607	89.6	11.8	6376.6	221.7
3966.360	72.4	13.1	5332.4	229.5
3971.958	26.1	5.2	6482.3	77.4
3980.311	1603.7	291.0	8027.2	3511.8
4136.214	45.6	9.8	6590.2	145.2

Table S3. MALDI-TOF peak list of alkylated rhOSM tryptic digest.
rhOSM was reduced with dithiothreitol and alkylated with iodoacetamide prior to tryptic digestion.

m/z	Peak Intensity	Signal to Noise Ratio	Peak Resolution	Peak Area
618.149	151.3	7.3	3696.8	38.2
731.252	175.2	8.4	4525.7	49.7
744.768	174.6	8.3	4780.0	51.6
782.422	4165.4	198.9	5640.4	958.2
833.357	993.5	47.3	5575.7	253.5
839.455	162.2	7.7	4886.9	50.5
844.407	1526.0	72.8	5751.8	384.8
869.462	106.9	5.1	4773.5	34.2
889.482	5912.0	281.2	6046.3	1532.8
898.548	340.3	16.2	5799.4	93.4
929.445	1953.8	93.5	5808.2	563.7
1117.561	178.5	8.4	6337.3	63.5
1177.505	282.5	13.3	5850.6	119.9
1194.538	359.7	16.9	5852.1	157.2
1203.595	166.4	7.8	6860.0	62.1
1258.805	175.7	8.3	1468.1	334.9
1365.558	297.7	14.0	1639.5	658.1
1368.023	176.6	8.3	4361.8	133.9
1381.797	8997.9	422.6	6987.6	4182.4
1454.896	122.3	5.7	2919.4	158.5
1470.694	132.1	6.2	6685.5	73.2
1473.966	263.3	12.4	2570.7	438.7
1475.949	171.4	8.1	11907.3	53.1
1488.726	15510.1	730.0	7373.1	7857.8
1727.811	6251.8	307.6	7833.3	3982.1
1742.897	217.1	10.7	7267.3	155.3
1764.349	156.0	8.1	3109.1	297.8
1784.938	396.4	21.6	3771.2	691.4
1787.077	246.9	12.3	9511.5	151.7
1796.185	116.6	6.0	1860.4	338.4
1799.942	66567.9	3338.7	6360.4	57246.0
1815.880	86.1	5.2	2516.2	225.9
1821.933	435.4	22.0	6505.0	371.9
1832.116	306.8	15.6	2426.6	710.1
1837.881	104.7	5.3	5738.3	112.1
1845.940	216.6	11.0	7578.0	165.0
1856.975	939.2	48.0	8053.9	674.1

1920.392	83.7	5.3	3786.4	166.5
1942.194	353.6	19.7	4623.4	480.0
1956.057	8952.3	505.6	7563.6	7273.2
2013.066	93.4	5.6	7289.5	81.5
2298.000	435.3	34.4	8064.9	406.3
2337.024	95.6	7.8	6669.2	117.8
2480.323	88.8	8.3	7187.1	111.1

Protein long-term stability assay

To identify buffer conditions that would promote OSM stability and prevent autolysis, OSM samples (33 μM) were stored at room temperature in 50 mM sodium phosphate, 100 mM sodium chloride, pH 6.6 with different buffer additives and assessed for degradation by SDS-PAGE analysis at 1 and 2 weeks.

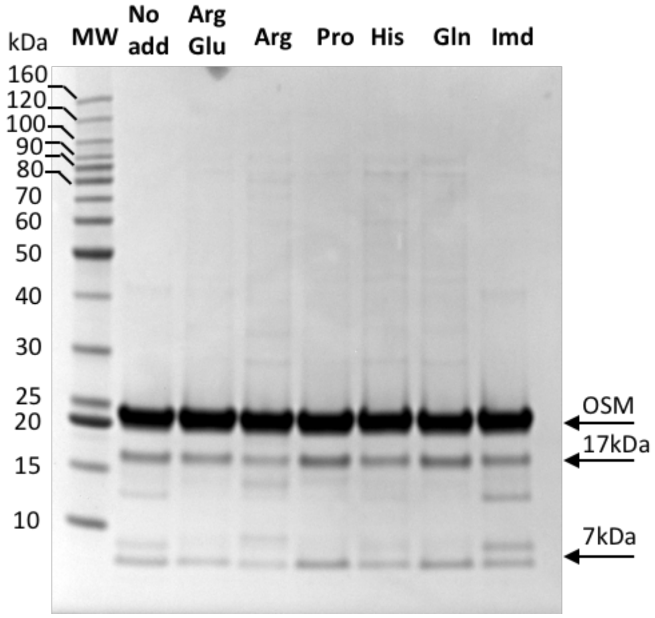

Figure S15. Effect of additives on long-term rhOSM stability.
SDS-PAGE of rhOSM stored for two weeks at room temperature in 50 mM $NaPO_4$, 100 mM NaCl, pH 6.6 with different additives. Prominent degradation products are indicated at 17 and 7 kDa. *Lane labels*: MW – MW ladder; No add – no additive; Arg/Glu –50 mM L-arginine/50 mM L-glutamic acid; Arg – 50 mM L-arginine; Pro – 100 mM proline; His – 15 mM L-histidine; Gln – 100 mM L-glutamine; Imd – 5 mM imidazole.

Chemistry

General Methods. All solvents and chemical reagents were obtained from commercial suppliers and used without further purification, unless otherwise stated. 4,5- dibromofuran-2-carbaldehyde (Compound 1), was purchased from Small Molecules, Inc. or was prepared according to the procedure of Chiarello et al.[5] and 3,4-methylenedioxyphenyl boronic acid was obtained from Combi-Blocks. N,N-Dimethylformamide (DMF) was distilled from P_2O_5 and stored over activated 3 Å molecular sieves. All glassware used in reactions was oven-dried (140°C) and cooled in a desiccator, fitted with septa and purged with anhydrous nitrogen immediately prior to use, unless otherwise indicated. All solvents and chemical reagents were transferred with a nitrogen-flushed syringe or cannula, unless otherwise indicated. ^{1}H and ^{13}C NMR spectra were obtained on either a Bruker Avance III Ultrashield Plus 600 MHz spectrometer or a Bruker Avance III Ultrashield 300 MHz spectrometer; chemical shifts of synthesized products were measured using residual solvent peaks as reference. High resolution mass spectrometry (HRMS) was conducted on a Bruker Daltonics maXis quadrupole time-of-flight spectrometer. IR data was obtained using a PerkinElmer FT-IR spectrometer with all samples analyzed using attenuated total reflection. Thin layer chromatography (TLC) was completed using 250 µm silica gel glass-backed plates with fluorescence indicator. Flash chromatography was completed using 230-400 mesh silica gel.

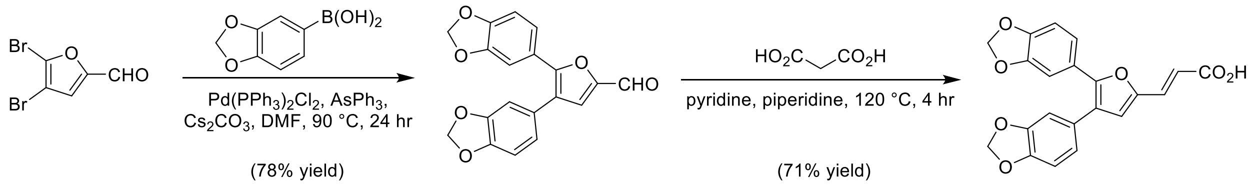

Scheme S1. Synthesis of OSM-SMI-10B

*4,5-bis(benzo[d][1,3]dioxol-5-yl)furan-2-carbaldehyde (**Compound 2)***

Compound 1 (0.990 g, 3.90 mmol) was dissolved in 26 mL dry, distilled DMF into which 3,4-methylenedioxyphenyl boronic acid (1.424 g, 8.58 mmol, 2.2 eq), cesium carbonate (7.624 g, 23.4 mmol, 6 eq), triphenylarsine (0.239 g, 0.78 mmol, 0.2 eq), and bis(triphenylphosphine)palladium(II) dichloride (0.382 g, 0.14 mmol, 0.16 eq) were added under a nitrogen atmosphere. The resulting brown solution was allowed to reflux at 90 °C for 24 hours and then concentrated under reduced pressure to remove excess DMF. The remaining residue was dissolved in 100 mL EtOAc and washed with 3 x 50 mL saturated $NaHCO_3$ solution. The organic layer was dried with $MgSO_4$, filtered, and concentrated to yield a brown solid. The crude product was applied to a 7 inch (5 cm) column containing silica gel eluted with 3:1 hexanes/EtOAc. Fractions containing pure product as determined by TLC (rf = 0.3, 3:1 hexanes/EtOAc) were combined and concentrated to afford 1.003 g of orange solid (78% yield). ^{1}H NMR ($CDCl_3$ with 0.03% v/v TMS, 600 MHz) δ: 9.63 (s, 1H), ,7.26 (s, 1H, overlap with $CDCl_3$), 7.19 (dd, 1H, J = 8.3 Hz, 1.7 Hz), 7.08 (d, 1H, J = 1.7 Hz), 6.85 (s, 2H), 6.82 (s, 1H), 6.78 (d, 1H, J = 8.2 Hz), 6.02 (s, 1H), 5.99 (s, 1H). ^{13}C NMR ($CDCl_3$ with 0.03% v/v TMS, 600 MHz) δ: 177.4, 154.0, 150.6, 148.8, 148.2, 148.0, 147.7, 127.3, 126.3, 125.1, 124.0, 123.4, 122.5, 122.2, 109.3, 109.0, 108.8, 107.6, 101.6, 101.5. HRMS m/z: $[M + H]^+$ Calcd for $C_{19}H_{12}O_6$ 337.0712; Found 337.0715, Error 0.89 ppm. IR: 2919, 2853 cm^{-1}.

*(E)-3-(4,5-bis(benzo[d][1,3]dioxol-5-yl)furan-2-yl)acrylic acid (**OSM-SMI-10B)***

A solution of aldehyde **2** (0.718 g, 2.14 mmol, 1.0 eq.), malonic acid (0.314 g, 3.02 mmol, 1.4 eq.), and a catalytic amount of piperidine (160 µL, 1.62 mmol, 0.75 eq.) in pyridine (3.2 mL) is heated to reflux (120 °C) under an N_2 atmosphere. Reaction is maintained under reflux until the evolution of CO_2 gas ceases (4 hr.), upon which the mixture is cooled to 0 °C and poured into ice cold 6M HCl with vigorous stirring. and the precipitate is collected

by suction filtration, washed 3 x 50 mL DI H_2O, and dried by suction filtration to afford 0.575 g of an orange solid (71% yield) that is greater than 95% pure, as determined by HPLC. ^1H NMR (600 MHz, DMSO-d6) δ 12.43 (s, 1H), 7.38 (d, *J* = 15.7 Hz, 1H), 7.06 (d, *J* = 9.8 Hz, 3H), 6.95 (dd, *J* = 15.4, 8.0 Hz, 2H), 6.91 (s, 1H), 6.85 (d, *J* = 8.0 Hz, 1H), 6.32 (d, *J* = 15.7 Hz, 1H), 6.06 (s, 2H), 6.05 (s, 2H). ^{13}C NMR (151 MHz, DMSO-d6) δ 167.5, 149.4, 148.5, 147.6, 147.5, 146.9, 130.2, 126.4, 123.6, 123.5, 122.0, 120.7, 119.4, 116.4, 108.8, 108.8, 108.7, 106.4, 101.5, 101.3. HRMS m/z: $[M + H]^+$ Calcd for $C_{21}H_{14}O_7$ 379.0812; Found 379.0815, Error 0.79 ppm. IR: 2902 cm^{-1}.

Characterization Data

- Compound 2
  - ^1H NMR Spectrum (full and expansions)
  - ^{13}C NMR Spectrum
- OSM-SMI-10B
  - ^1H NMR Spectrum (full and expansions)
  - ^{13}C NMR Spectrum
  - HRMS
  - HPCL Chromatogram

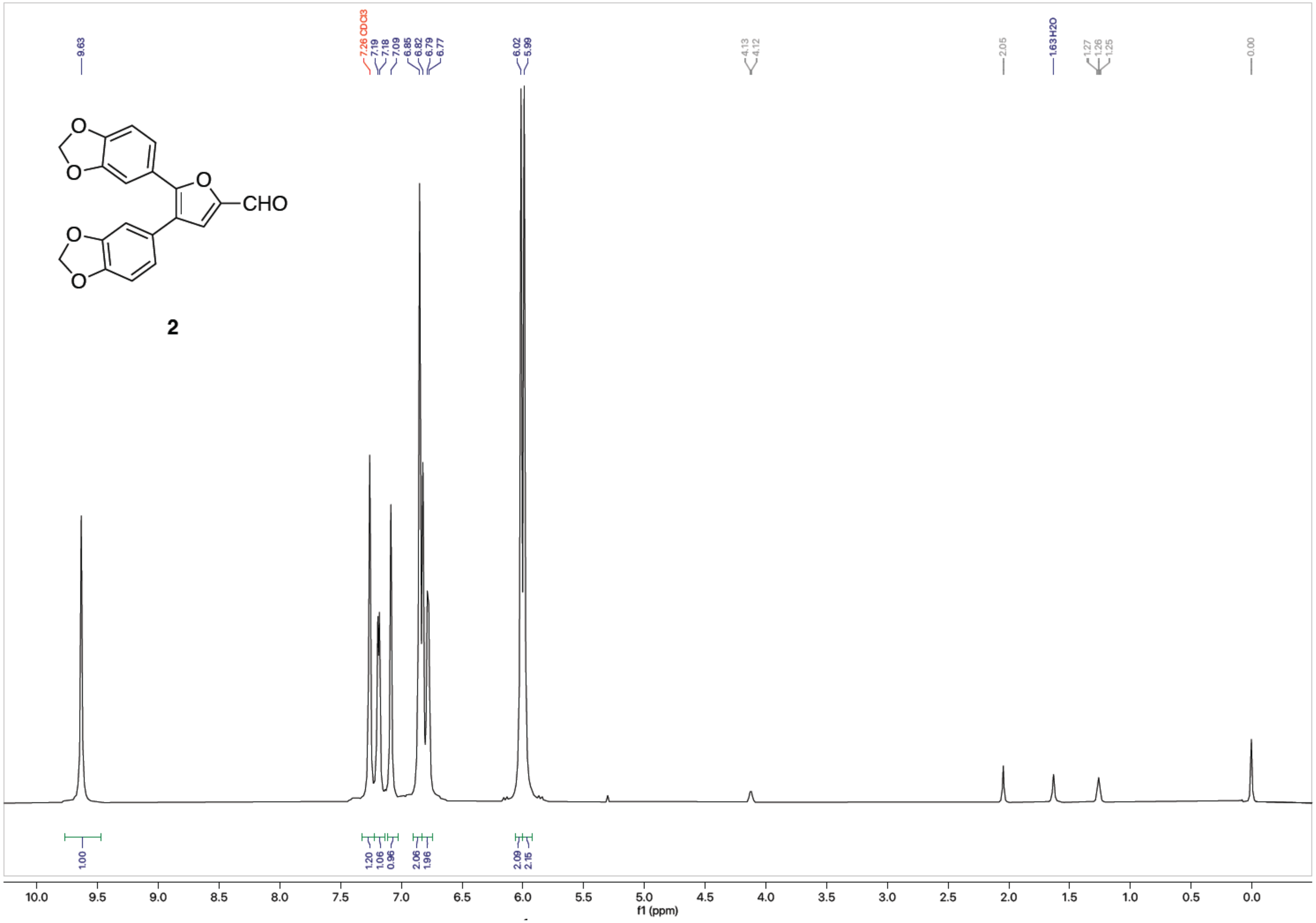

Figure S16. ^1H **NMR (600 MHz,** $CDCl_3$**) aldehyde 2**

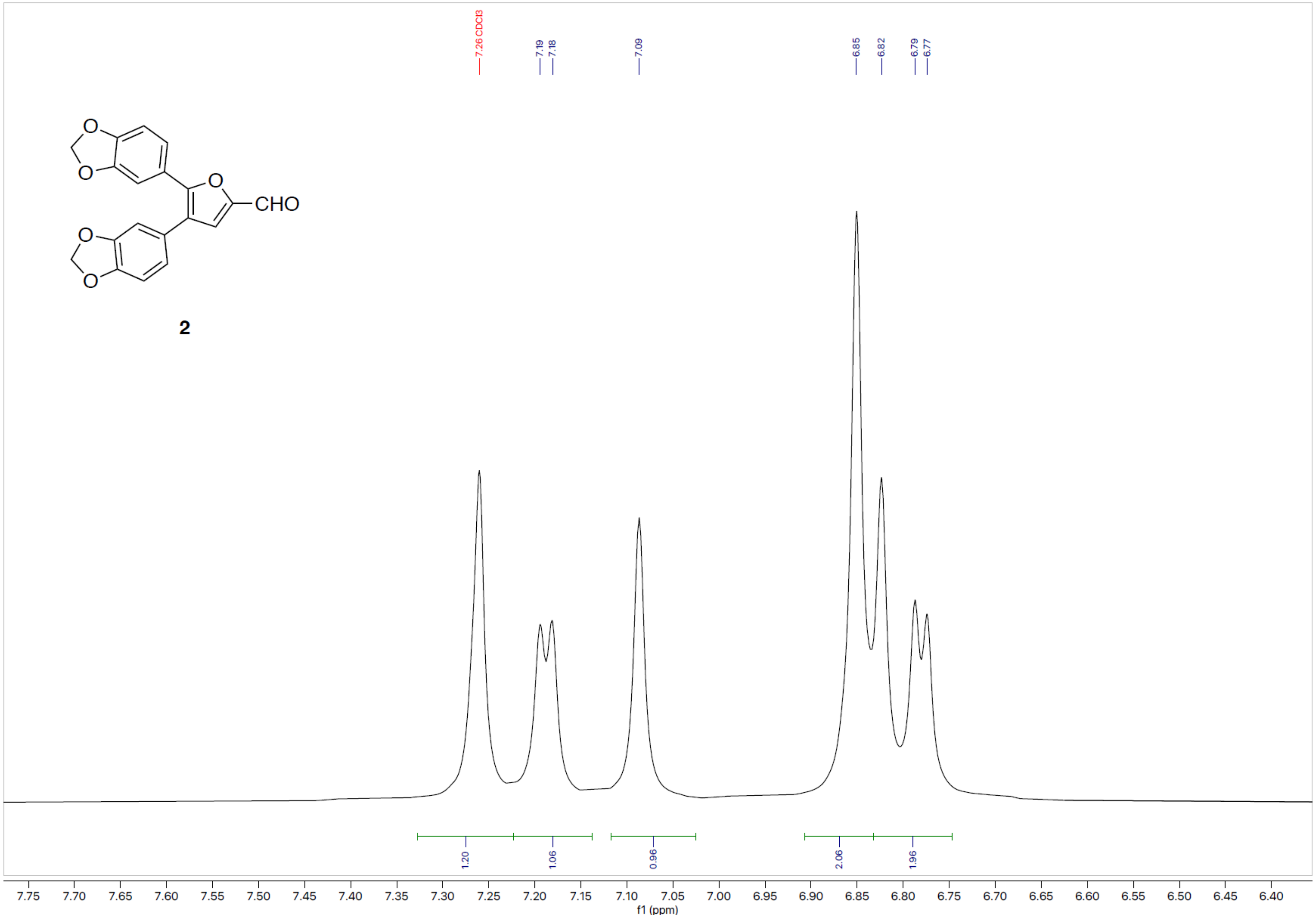

Figure S17. ^1H **NMR (600 MHz,** $CDCl_3$**) aldehyde 2 (inset)**

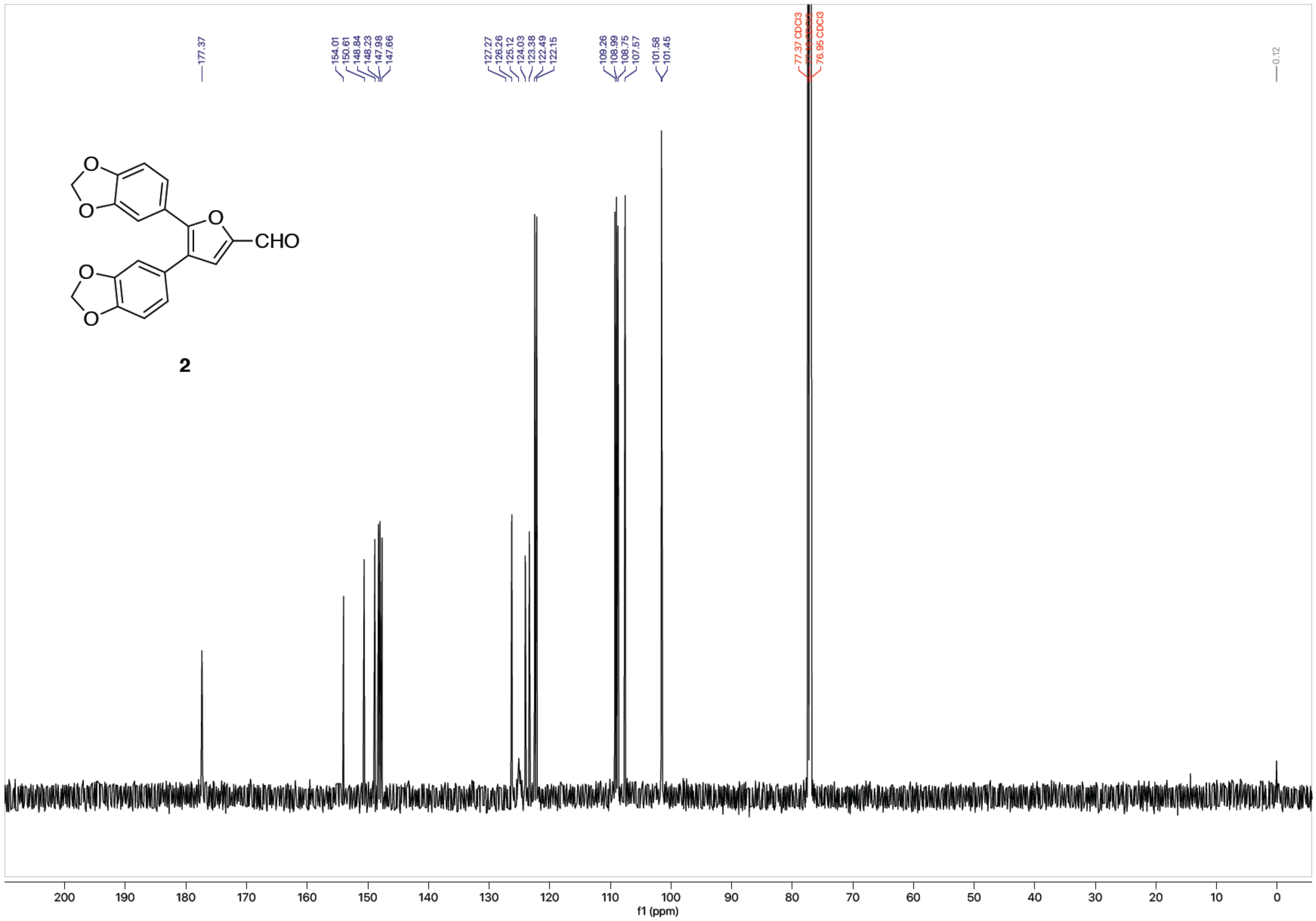

Figure S18. ^{13}C NMR (151 MHz, $CDCl_3$) aldehyde 2

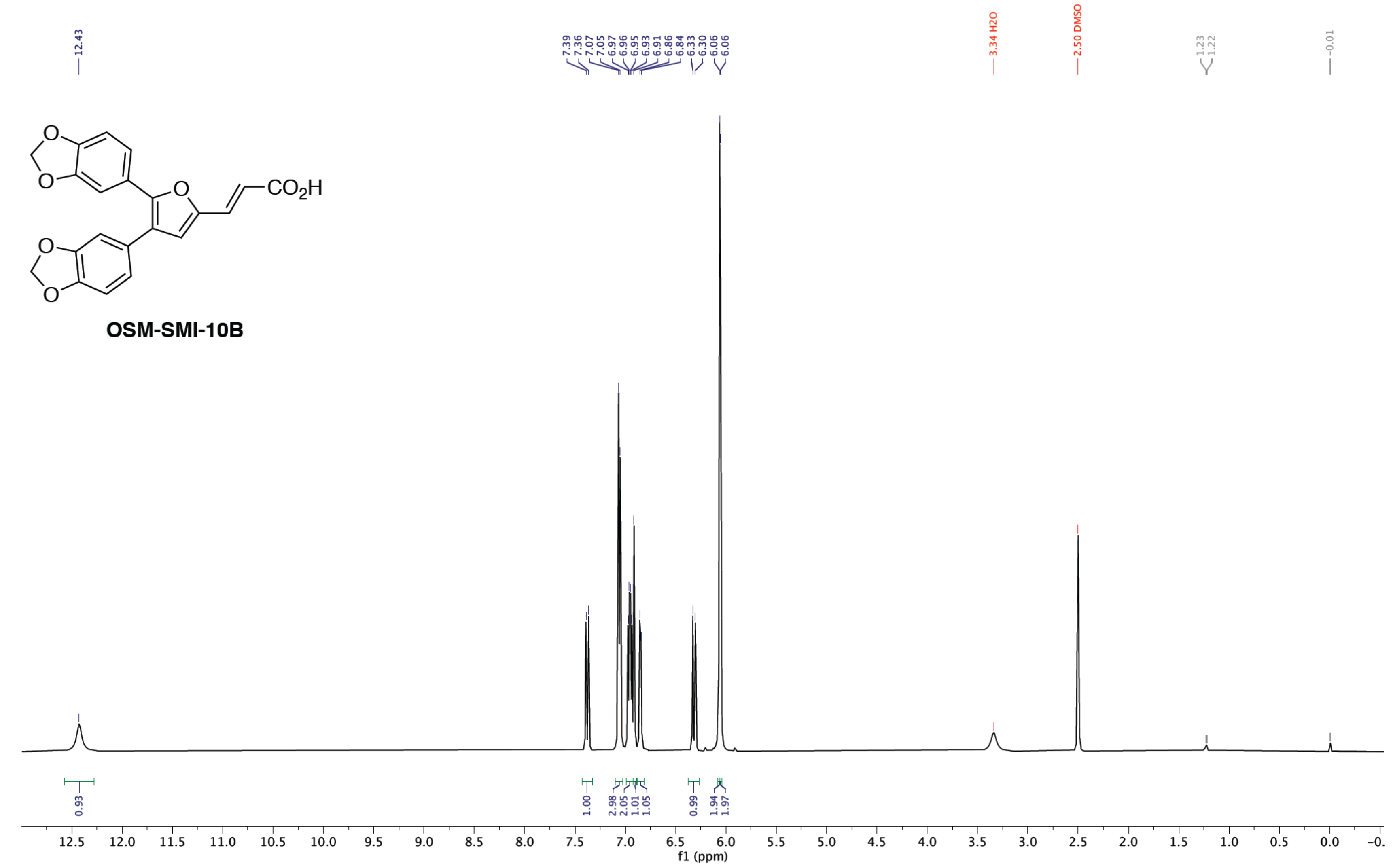

Figure S19. ^{1}**H NMR (600 MHz, DMSO-d6) OSM-SMI-10B**

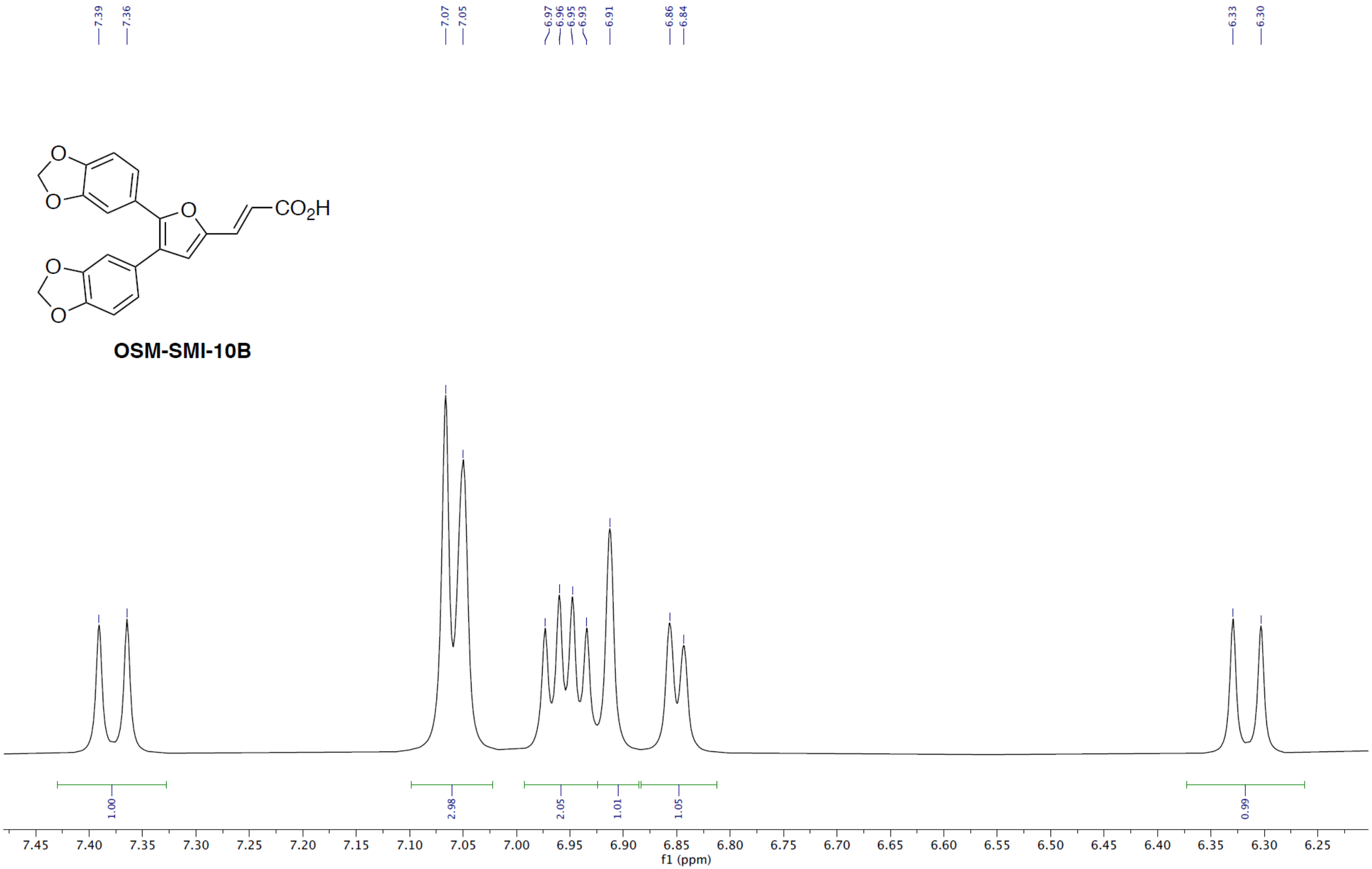

Figure S20. ^{1}**H NMR (600 MHz, DMSO-d6) OSM-SMI-10B (inset).**

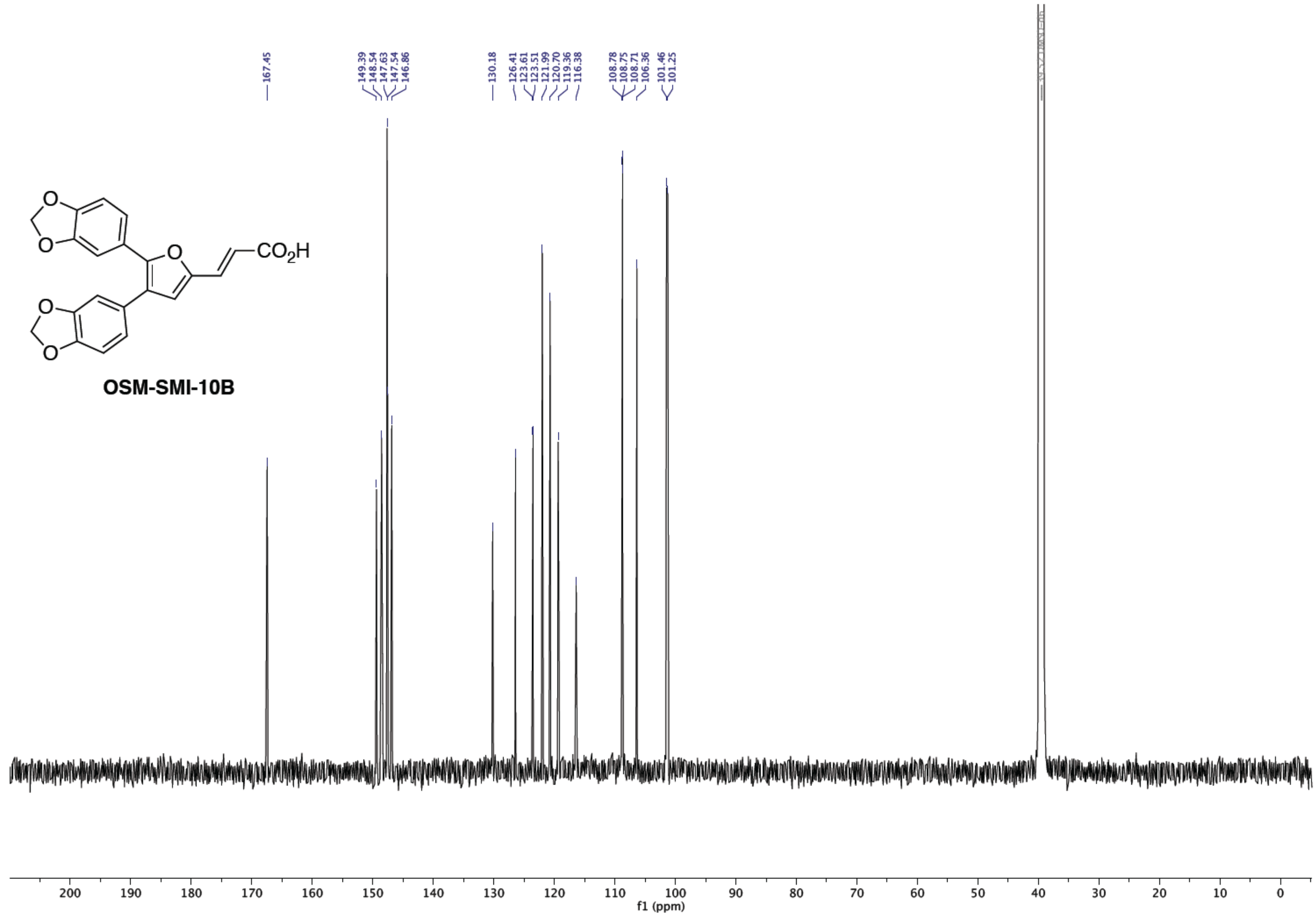

Figure S21. ^{13}C **NMR (600 MHz, DMSO-d6) OSM-SMI-10B**

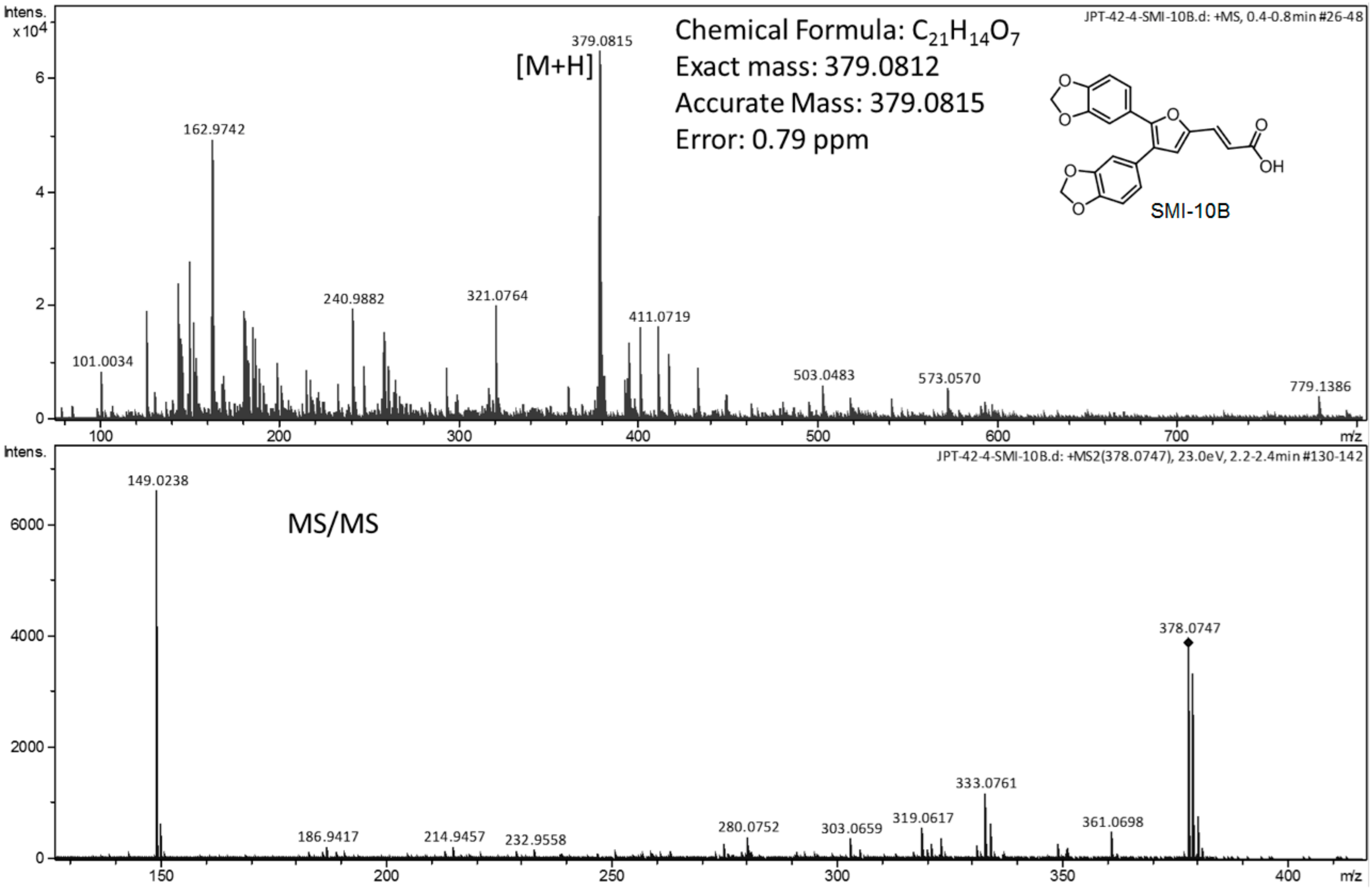

Figure S22. HRMS and MS/MS of $[M+H]^+$ of OSM-SMI-10B

```
=====================================================================
Acq. Operator   : Tyson                          Seq. Line :   1
                                                  Location : Vial 1
Injection Date  : 20-Sep-19, 13:14:27                  Inj :   1
                                                Inj Volume : 3.0 µl
Acq. Method     : JPT-DW-10B.M
Analysis Method : C:\STOCKROOM\METHODS\CLEANING METHOD.M
Last changed    : 2/18/2021 1:29:13 PM by Tyson
                  (modified after loading)
```

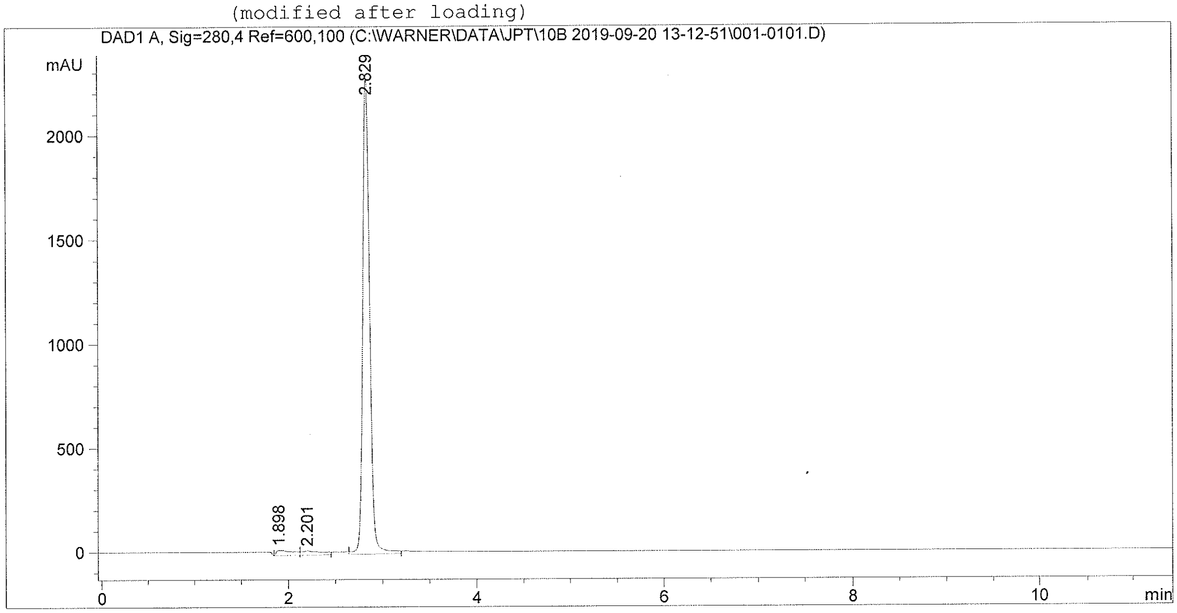


```
=====================================================================
                         Area Percent Report
=====================================================================

Sorted By             :      Signal
Multiplier:                    :      1.0000
Dilution:                      :      1.0000
Use Multiplier & Dilution Factor with ISTDs


Signal 1: DAD1 A, Sig=280,4 Ref=600,100
```

Peak #	RetTime [min]	Type	Width [min]	Area [mAU*s]	Height [mAU]	Area %
1	1.898	VV	0.1505	310.57739	25.78389	2.5373
2	2.201	VV	0.1902	322.04688	21.32768	2.6310
3	2.829	VV	0.0790	1.16080e4	2296.25488	94.8318

```
Totals :                     1.22406e4  2343.36645

=====================================================================
                         *** End of Report ***
```

Figure S23. HPLC Chromatogram of OSM-SMI-10B

Fluorescence Quenching

Fluorescence quenching was used as a method to detect and quantify SMI binding to OSM. The fluorophore, W187, is located at the C-terminus of rhOSM1-187, is the only Trp in the human OSM gene, and is distal to the proposed SMI binding site. In the crystal structure, W187 is packed in the middle of the 4-helix bundle and is in proximity of Tyr10, Arg67, Phe70, Leu 71, Met 128, and Phe184 (**Supplemental Fig. S16A**). In the presence of increasing SMI, the fluorescence decreases, indicating quenching of W187 (**Supplemental Fig. S16B**). Negative deviation from a linear Stern-Volmer plot of (f_0/f) obtained from OSM titrated with OSM-SMI-10B (**Supplemental Fig. S16C**) illustrates that the quenching of W191 is likely a result of indirect or allosteric effects with a fraction of the fluorophore accessible to quenching or only partial quenching.[4] We interpret this as slight allosteric structural changes due to binding of the SMI at the distal end of the 4-helix bundle (which is supported by the NMR titration data). However, regardless of the nature of the quenching of W187, linearity of the modified Stern-Volmer plot ($f_0/\Delta f$ vs. 1/[X]) (**Supplemental Fig. S16D**) demonstrates that the dissociation constant, K_d and the fraction of fluorophores available for quenching (f_a) may be calculated from the fluorescence quenching data.[4]

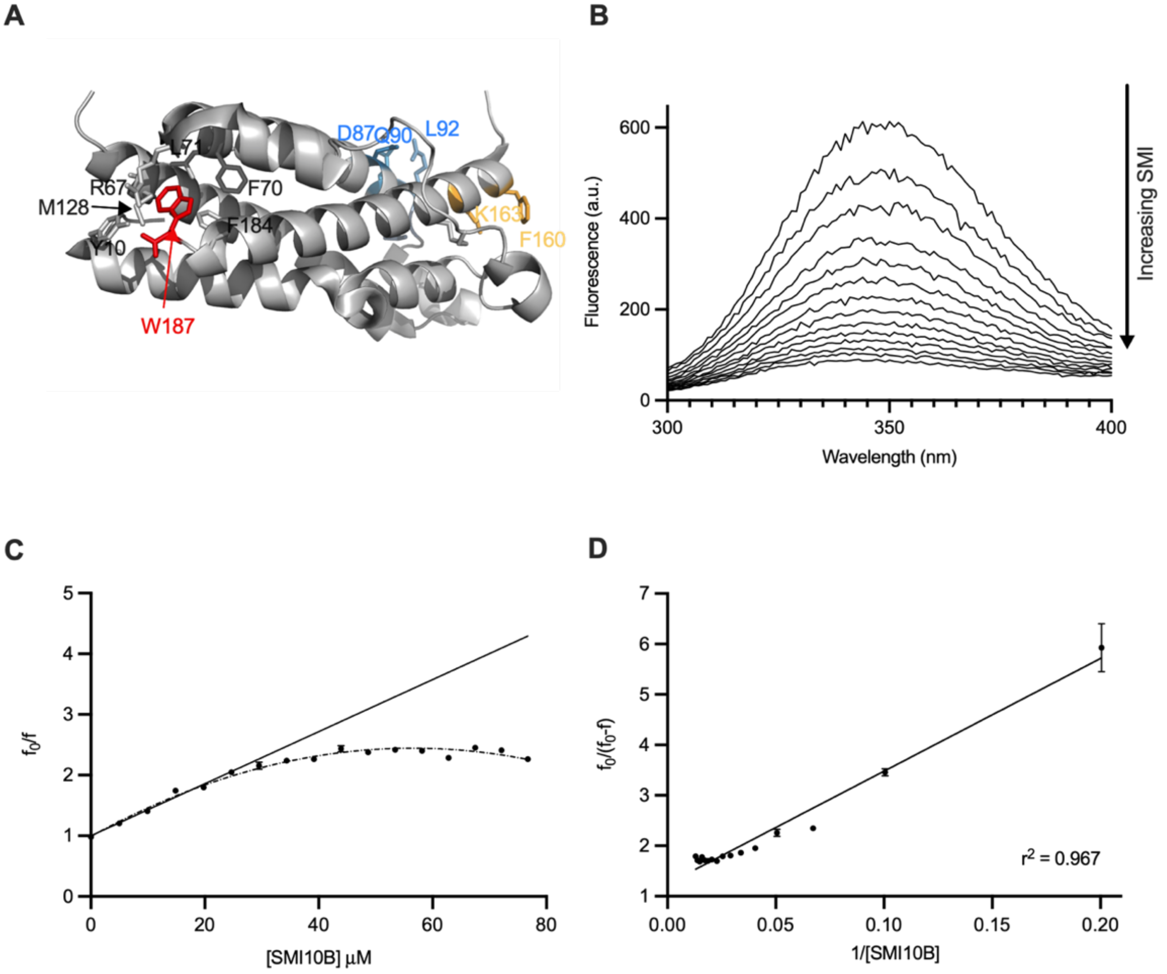

Figure S24. Quenching of rhOSM tryptophan 187 fluorescence by OSM-SMI-10B.
(**A**) Structure of hOSM with W187 highlighted in red. Residues within 5 A of W187 are shown as sticks and grey. Residues identified in the NMR titration are shown in gold and blue. (**B**) Fluorescence intensity plotted over the measured emission range from 345 – 355 nm with increasing concentration of OSM-SMI-10B. (**C**) Relative fluorescence intensity plotted as a function of OSM-SMI-10B concentration. The Stern-Volmer equation fit to the linear region of the plot (solid line) and a quadratic fit (dashed line) are shown. (**D**) Modified Stern-Volmer plot.

A rearrangement of the modified Stern-Volmer equation, $\frac{f}{f_0} = \frac{f_a}{1-\frac{[X]}{Kd}} + (1 - f_a)$, was fit to the fluorescence quenching data for SMI-10B (**Supplementary Fig. S17A**). A dissociation constant of 10.37 ± 0.81 µM and the fraction of accessible fluorophores, 0.70 ± 0.01, was determined from the fit. The K_d is consistent with the value determined from the NMR titration data.

A comparison of the NMR and fluorescence quenching experiments to two additional SMI compounds is shown in Supplementary Figure S18. OSM-SMI-10B11 is a derivative of OSM-SMI-10B and OSM-SMI-27A6 is

chemically unique SMI, synthesized based on QSAR and computational modelling (structured not shown). The binding affinities are similar between OSM-SMI-10B and OSM-SMI-10B11 and the CSPs have a similar pattern. OSM-SMI-27A6 is 2-fold weaker in affinity and shows fewer CSPs, although, due to solubility, the maximum titration point was only 80 µM. This comparison highlights the utility of NMR, but in conjunction with complementary methods.

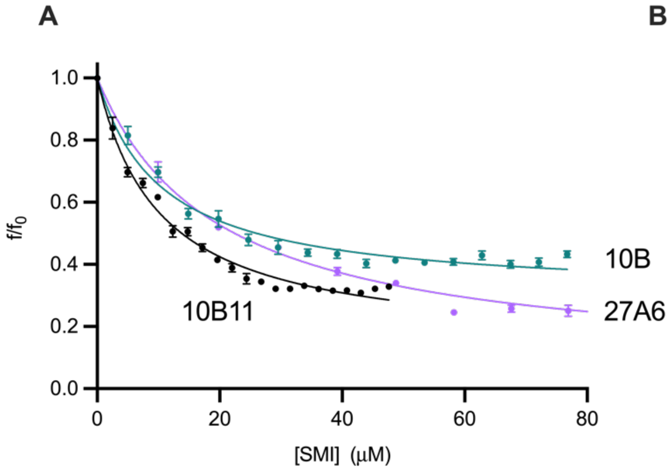

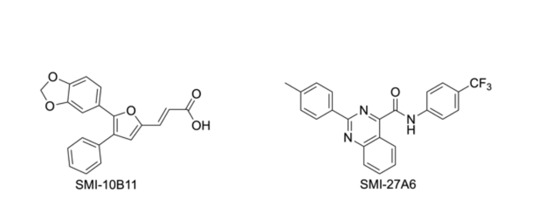

SMI	K_d	f_a
OSM-SMI-10B	10.37 ± 0.81	0.70 ± 0.01
OSM-SMI-10B11	10.10 ± 0.52	0.87 ± 0.01
OSM-SMI-27A6	19.56 ± 1.30	0.94 ± 0.02

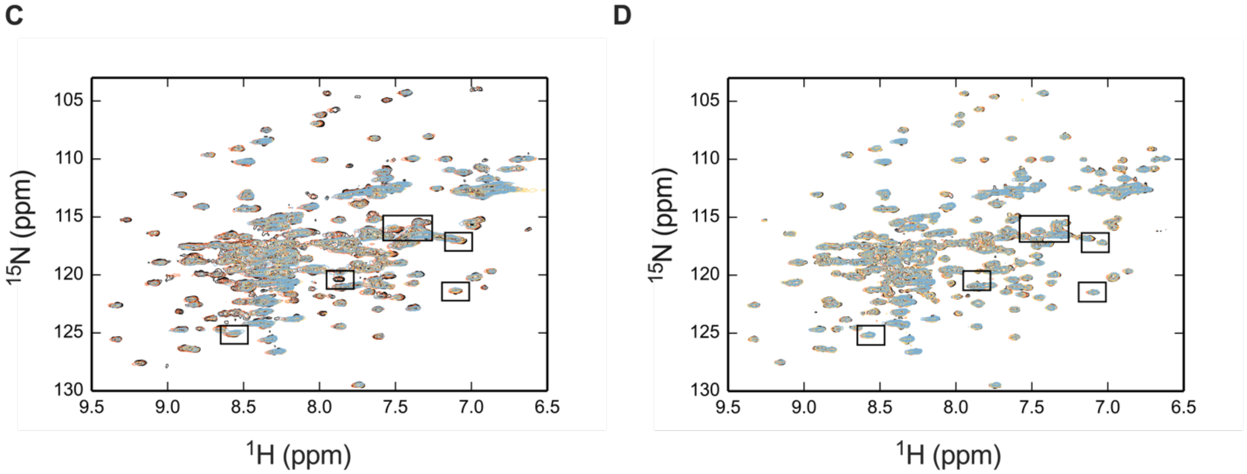

Figure S25. Comparison of fluorescence quenching and NMR titration data for OSM-SMI-10B11 and OSM-SMI-27A6 with OSM-SMI-10B.
(**A**) The quenching of W187 fluorescence for OSM-SMI's titrated into 1 µM of rhOSM.is plotted as f/f_0 and a modified version of the Stern-Volmer equation was fit to the data (solid line). (**B**) Structures of OSM-SMI-27A6 with OSM-SMI-10B. Binding affinities and fraction of accessible fluorophores fitted from the data in (**A**). (**C**) 100 µM of ^{15}N rhOSM titrated with OSM-SMI-10B11 up to 200 µM of SMI. Color ramped from orange to blue. Black boxes indicate peaks that shifted significantly in OSM-SMI-10B. (**D**) 100 µM of ^{15}N rhOSM titrated with OSM-SMI-27A6 up to 80 µM of SMI. Color ramped from orange to blue. Black boxes indicate peaks that shifted significantly in OSM-SMI-10B.

References

1. Baneyx, F. & Mujacic, M. Recombinant protein folding and misfolding in Escherichia coli. *Nature Biotechnology* (2004). doi:10.1038/nbt1029

2. Pryor, K. D. & Leiting, B. High-level expression of soluble protein in Escherichia coli using a His6-Tag and maltose-binding-protein double-affinity fusion system. *Protein Expr. Purif.* (1997). doi:10.1006/prep.1997.0759

3. Prasad, S., Khadatare, P. B. & Roy, I. Effect of Chemical Chaperones in Improving the Solubility of Recombinant Proteins in Escherichia coli †. *Appl. Environ. Microbiol.* **77**, 4603–4609 (2011).

4. Lakowicz, J. R. *Principles of fluorescence spectroscopy*. *Principles of Fluorescence Spectroscopy* (Springer, 2006). doi:10.1007/978-0-387-46312-4

5. Chiarello, J. & Joullié, M. M. Synthetic routes to cristatic acid and derivatives. *Tetrahedron* (1988). doi:10.1016/S0040-4020(01)85090-0
